# KRAS is a molecular determinant of platinum responsiveness in glioblastoma

**DOI:** 10.1186/s12885-023-11758-6

**Published:** 2024-01-15

**Authors:** Candida Zuchegna, Stefano Leone, Antonella Romano, Antonio Porcellini, Samantha Messina

**Affiliations:** 1 Department of Biology, Federico II University of Naples, 80126 Naples, Italy; 2 Department of Epidemiology, Preclinical Research and Advanced Diagnostics, National Institute for Infectious Diseases IRCCS ‘L. Spallanzani’, Rome, Italy; 3 Department of Anatomical, Histological, Forensic Medicine and Orthopedic Sciences, Sapienza University of Rome, 00161 Rome, Italy; 4 Department of Science, Roma Tre University, Viale Guglielmo Marconi 446, 00146 Rome, Italy

**Keywords:** *KRAS*, Glioblastoma, Cisplatin, Chemosensitivity, MEK-inhibitor

## Abstract

**Background:**

*KRAS* is the undisputed champion of oncogenes, and despite its prominent role in oncogenesis as mutated gene, KRAS mutation appears infrequent in gliomas. Nevertheless, gliomas are considered KRAS-driven cancers due to its essential role in mouse malignant gliomagenesis. Glioblastoma is the most lethal primary brain tumor, often associated with disturbed RAS signaling. For newly diagnosed GBM, the current standard therapy is alkylating agent chemotherapy combined with radiotherapy. Cisplatin is one of the most effective anticancer drugs and is used as a first-line treatment for a wide spectrum of solid tumors (including medulloblastoma and neuroblastoma) and many studies are currently focused on new delivery modalities of effective cisplatin in glioblastoma. Its mechanism of action is mainly based on DNA damage, inducing the formation of DNA adducts, triggering a series of signal-transduction pathways, leading to cell-cycle arrest, DNA repair and apoptosis.

**Methods:**

Long-term cultures of human glioblastoma, U87MG and U251MG, were either treated with cis-diamminedichloroplatinum (cisplatin, CDDP) and/or MEK-inhibitor PD98059. Cytotoxic responses were assessed by cell viability (MTT), protein expression (Western Blot), cell cycle (PI staining) and apoptosis (TUNEL) assays. Further, gain-of-function experiments were performed with cells over-expressing mutated hypervariable region (HVR) KRAS^G12V^ plasmids.

**Results:**

Here, we studied platinum-based chemosensitivity of long-term cultures of human glioblastoma from the perspective of *KRAS* expression, by using CDDP and MEK-inhibitor. Endogenous high KRAS expression was assessed at transcriptional (qPCR) and translational levels (WB) in a panel of primary and long-term glioblastoma cultures. Firstly, we measured immediate cellular adjustment through direct regulation of protein concentration of K-Ras4B in response to cisplatin treatment. We found increased endogenous protein abundance and involvement of the effector pathway RAF/MEK/ERK mitogen-activated protein kinase (MAPK) cascade. Moreover, as many MEK inhibitors are currently being clinically evaluated for the treatment of high-grade glioma, so we concomitantly tested the effect of the potent and selective non-ATP-competitive MEK1/2 inhibitor (PD98059) on cisplatin-induced chemosensitivity in these cells. Cell-cycle phase distribution was examined using flow cytometry showing a significant cell-cycle arrest in both cultures at different percentage, which is modulated by MEK inhibition. Cisplatin-induced cytotoxicity increased sub-G1 percentage and modulates G2/M checkpoint regulators cyclins D1 and A. Moreover, ectopic expression of a constitutively active KRAS^G12V^ rescued CDDP-induced apoptosis and different HVR point mutations (particularly Ala 185) reverted this phenotype.

**Conclusion:**

These findings warrant further studies of clinical applications of MEK1/2 inhibitors and KRAS as ‘actionable target’ of cisplatin-based chemotherapy for glioblastoma.

**Supplementary Information:**

The online version contains supplementary material available at 10.1186/s12885-023-11758-6.

## Background

Glioblastoma (GBM) is an incurable cancer type [27]. Primary glioblastomas (GBMs) are often associated with disturbed RAS signaling, although mutations in *KRAS* gene are rare in human gliomas and particularly rare in WHO grade III and IV gliomas in adult patients [21, 26, 32, 40]. Nevertheless, glioblastoma is considered KRAS-driven cancer due to its essential role in mouse malignant gliomagenesis [14, 17, 18, 36]. Although RAS alterations are not commonly reported in GBMs [21, 23, 45, 46], GBM possesses mutations in genes that contribute to activated KRAS signaling, like neurofibromin-1 (NF1), are observed, which make KRAS signaling a potential target in GBM [3, 5]. More recently, genomic characteristics of cerebellar glioblastoma C-GBMs reported *RAS* hotspot mutation or amplification [8]. Targeting glioblastoma (GBM) based on molecular subtyping has not yet translated into successful therapies.

Combinatorial therapy based on temozolomide (TMZ) and cisplatin (CDDP) shows promising potential for GBM therapy in clinical trials [43]. Cisplatin is the mainstay in cancer chemotherapy for multiple tumour types, including medulloblastoma but not glioblastoma protocols. There is no clear explanation for the differences in clinical efficacy of cisplatin between medulloblastomas and glioblastomas, even though cisplatin is effective in vitro against the latter. Although cisplatin has been shown to have cytotoxic effects on human glioblastoma cells in vitro [20, 41] the response in clinical treatment is weak and has not improved the overall survival of patients with brain tumours. Anyway, many studies are currently focused on new delivery modalities of effective cisplatin in GBM [2, 6, 37, 43]. The mechanism of action of cisplatin is mainly based on DNA damage, inducing the formation of DNA adducts. The DNA lesions trigger a series of signal-transduction pathways, leading to cell-cycle arrest, DNA repair and apoptosis [13].

Within the area of combinatorial therapies, MEK inhibitors have currently growing advances in clinical trial in glioblastoma treatment [19]. PD98059, a potent but reversible MEK inhibitor, has recently developed as a new formulation to obtain long-term inhibition of pERK1/2 in brain regions at detectable levels [30]. PD98059 belongs to the first-generation MEK1/2 inhibitors, its inhibitory properties by binding to the ERK-specific MAP kinase MEK, therefore preventing phosphorylation of ERK1/2 (p44/p42 MAPK) by MEK1/2. PD98059 does not inhibit the MAPK homologues JNK and P38 [7, 12]. Unfortunately, despite wide use in preclinical studies, this compound failed to reach clinical evaluation because of its pharmaceutical limitations [24, 28]. In general, targeting MEK and other downstream proteins in the RAS signaling cascade has shown limited efficacy in RAS-driven malignancies, likely owing to dose-limiting toxicity and loss of auto-inhibitory feedback.

Cyclin D1 is a cell-cycle regulator essential for G1, phase progression and a candidate proto-oncogene implicated in pathogenesis of several human tumour types, including glioblastomas [44]. Cyclin D1, in association with CDK4/6, acts as a mitogenic sensor and integrates extracellular mitogenic signals and cell cycle progression. When deregulated (overexpressed, accumulated, inappropriately located), cyclin D1 becomes an oncogene and is recognized as a driver of solid tumours. Cyclin D1 (CCND1) is upregulated in many solid cancers, promoting cancer progression [29, 38]. Cyclin D1 expression has been shown to be associated with the pathological grade and aggressiveness of glioma, the prognosis of patients with glioma, and the response to chemotherapy [22, 33, 42].

In this study, we used cis-diamminedichloroplatinum (cisplatin, CDDP) to treat long-term cultures of human malignant glioblastoma to study their KRAS- dependent chemosensitivity. Gain-of-function experiment with constitutively active KRAS^G12V^ enhanced glioblastoma sensitivity to CDDP measured by apoptosis and viability; interestingly, specific post-translational modification in HVR region altered sensitivity to cisplatin and/or MEK inhibition. The aim of our study was to elucidate the relationships between KRAS and its post-translational modification, MEK-inhibitor, and cancer cell response to chemotherapy with cisplatin in vitro.

## Methods

### Long-term and primary glioblastoma cultures

Human glioblastoma cell lines U87MG, U251MG, T98G, IDH1^mut^U87, SW1783, and Ln229 were obtained from the American Type Culture Collection, Rockville, MD and cultured at 37 °C in 5% CO_2_ in Dulbecco's Modified Eagle’s Medium (DMEM), supplemented with phenol red, L-glutamine (2 mM), 1% pen-strep and 10% Fetal Bovine Serum (FBS; South America origin, Brazil). SVGp12 Human Fetal Glial Cells (ATCC-CRL-8621 # 4282167) and NHA cells (purchased from Cambrex Corporation, East Rutherford, NJ) were grown according to the manufacturer’s instructions. Normal Primary Fetal Normal Neural Stem Cells from SVZ neural stem cells were derived from brain subventricular zone (SVZ) tissue of a premature neonate died of pulmonary failure; the continuous culture from this tissue is indicated as SC-30 (SC30, 25-week gestation, 1-day-old premature infant; [34].—Astrocytoma primary (WHO grade IV,samples GBM#C; GBM#D; GBM#F; GBM#M were established from tumor specimens of patients and cultured as described [25, 47], . Astrocytoma primary (WHO grade IV) GBM#1; GBM#10; GBM#107; GBM#11; GBM#148; GBM#15; GBM#47; GBM#53; GBM#80; GBM#82 were established from tumor specimens of patients and cultured as described [31]. The genetic background of U87MG and U25MG1 long-term cultures are: U87MG (p53 wild type, IDH1 w.t.; low level of methyl guanine transferase (MGMT) cells; U251MG (p53 mutated; IDH1 w.t.; low level of methyl guanine transferase (MGMT). Moreover, U87MG is highly cytogenetically aberrant [10, 15].

### RT PCR and qPCR

Total RNA was extracted using the acid guanidinium isothiocyanate-phenol–chloroform method. cDNA was synthesized in 20-μl reactions containing 2 μg of total RNA, 200 units of Superscript III Reverse Transcriptase (Invitrogen), and 1 μl of random hexamer (20 ng/μl) (Invitrogen). mRNA was reverse-transcribed for 1 h at 50 °C, the reaction was heat-inactivated for 15 min at 70 °C. The products were stored at -20 °C until use. Quantitative (q) RT-PCR were performed on an Applied Biosystems ABI StepOne Plus Real-Time PCR 96-Well System using the SYBR Green-detection system (FS Universal SYBR Green MasterRox/Roche Applied Science). For all reactions, following conditions were used: 95 °C for 10 min, 40 cycles of 95 °C for 30 s and 58 °C for 75 s. Primer sequences used are listed below: KRAS F: 5’ – TTG CCT TCT AGA ACA GTA GAC A – 3’ KRAS R: 5’ – TTA CAC ACT TTG TCT TTG ACT TC – 3’. Fold changes were normalized against the reference gene (18 S) amplified with the following primer sets: 18S F: 5’ – GAC CGA TGT ATA TGC TTG CAG AGT—3’; 18S R: 5’ – GGA TCT GGA GTT AAA CTG GTC CAG – 3’.

### Antibodies and reagents

Monoclonal anti-panRas antibody (Ab-3) was purchased from Calbiochem (EMD Biosciences, an Affiliate of Merck KGaA, Darmstadt, Germany); The antibodies against Ki-Ras (sc-521), anti-ERK, phospho-ERK, anti-cyclin A, anti-p53, anti-cyclin D1, anti-p27 and anti-p53 were from Santa Cruz Biotechnology (Santa Cruz, CA, USA). The anti-β- actin was from Sigma–Aldrich (St. Louis, MO, USA). The peroxidase-conjugated (HRP) anti-rabbit, anti-mouse secondary antibodies, nitrocellulose membrane PROTRAN and the ECL detection system were from Amersham-Pharmacia (Biothec, UK Limited). Fetal bovine serum (FBS), trypsin–EDTA, and penicillin/streptomycin solutions were purchased from HyClone Europe Ltd. (Cramlington, UK); Dulbecco’s Modified Eagle’s Medium (DMEM) and Lipofectin reagent were from GIBCO BRL, Life Technologies (Carlsbad, CA, USA). All other reagents were purchased from Sigma Aldrich (Milano, Italy).

### Western blot assay

Cells were exposed to cisplatin (CDDP) 16,6 µM and/or to the MEK-inhibitor PD98059 40 μM for the indicated times as described in the figures’ legends, harvested at times indicated, and lysed on ice-cold RIPA buffer (1% Triton X-100, 0.5% DOC, 0.1% SDS, 50 mM Tris–HCl, pH 7.6, 150 mM NaCl, 1 mM PMSF, and 1 mg/ml aprotinin, leupeptin, and pepstatin). After centrifugation at 12,000 g, protein concentrations were determined by Bradford assay. Twenty to fifty micrograms of protein were subjected to 7% or 12% SDS–PAGE and transferred onto nitrocellulose membranes (Schleicher & Schuell, Germany). Blots were then blocked in Tris-buffered saline (50 mM TrisHCl, 200 mM NaCl, pH 7.4) containing 5% nonfat dry-milk (Bio-Rad Laboratories Inc.,Hercules, CA) and incubated with primary antibodies as follows: anti-pan-Ras antibody, Ab-3, 1: 500; anti-Ki-Ras antibody, 1: 200; anti-Ha-Ras antibody, 1: 400, all incubated overnight at 4◦C; anti-β-actin, 1: 1,000, incubated 2 h at room temperature; anti-ERK1/2 and anti-phospho-ERK1/2 (1: 1,000), incubated 2 h at room temperature). Blots were washed three times with PBS and then incubated for 2 h with horseradish peroxidase-conjugated secondary antibodies (all used at 1: 5,000). Immunostaining was revealed by the ECL detection system (Amersham).

### Cell transfection

Human U251MG, U87MG and HEK-293 cells were transiently transfected with Ras constructs mutant at carboxyl-terminal hypervariable region (HVR): (1) constitutively active K-Ras carrying a Val-12 point mutation (KRAS4B V12: Val 12 constitutively active instead of Gly; (2) a double K-Ras mutant carrying Val-12 and Ala-185 mutations (KRAS4B V12A185: Ala instead of Cys > prevent farnesylation); (3) a triple K-Ras mutant carrying Val-12, Glu-177 and Ala-185 mutations (KRAS4B V12E177 E = Glu E177 instead of Lys > disrupt KKKKK Lysine stretch); (4) a double mutant H-Ras carrying Leu-61 and Ser-186 mutations (HRASL61S186, Leu-61, Ser-186; it is a cytoplasmic, GTP-bound interfering Ras mutant [48]. HEK-293 cells were plated onto 100-mm Falcon dishes and grown in DMEM containing 10% FBS. One day after plating, cells were transfected with 10 μg of cDNA in serum free medium using a Lipofectin™ Transfection Reagent (Thermo Fisher Scientific Inc.), according to manufacturer’s instructions. Two hours later, cultures were switched into the growing medium. After 24 h, the cells were then processed for fluorescence-activated cell sorting (FACS) analysis. Complete sequence verification of the DNA plasmids carrying point mutations were assessed by a modification of the Sanger dideoxy method as implemented in a double stranded DNA cycle sequencing system with fluorescent dyes. Sequence reactions were then run on a 3130 Automated sequence system (Applied Biosystem) [1].

### Cell cycle distribution analysis

Flow cytometry was used to determine the cell cycle distribution using a cell cycle kit with PI staining (BD Biosciences). U87MG and U251MG cells were plated in 6-well plates and treated with various concentrations (0, 1, 2, 5 µM) of cisplatin for 72 h. Then, the cells were collected by centrifugation at 167.7 × g for 5 min at room temperature. Subsequently, the cells were washed and fixed with PBS and cold 70% ethanol for 24 h 4 °C. Then, the cells were treated with 50 µl 100 µg/ml RNase at 37 °C, washed twice with PBS, centrifuged at 167.7 × g for 5 min and stained with 5 µl PI (50 mg/ml stock solution). The results were analyzed by BD FACSAria (BD Biosciences). The data were quantified using ModFit LT 4.0 (Verity Software House, Inc.) [25].

### TUNEL assay

5 × 10^5 cells were grown in 60 mm dishes. At 18 h after treatment, cells were fixed in 2% paraformaldehyde/1 × PBS for 10 min at RT and washed once in PBS þ 50 mM glycine for 10 min at RT and washed again three times for 5 min in PBS. Cells were permeabilized with 0.5% Triton X-100/1 × PBS for 10 min, washed 3 × 5 min in PBS and incubated with 100 µl of 1 × TdT reaction mix. TdT-mediated dNTP nick end labeling was carried out at 37°C for 60 min using 15 U of TdT (Roche Diagnostics S.p.A, Roche Applied Science, Monza, Italy) and 2 µl of 2 mM BrdUTP. BrdUTP incorporation was revealed by anti-BrdU-FITC and then stained with propidium iodide. The data were acquired and analysed by CELLQuest software for bivariate analysis of DNA content versus BrdU. Experiments were performed in triplicate [11].

### Viability assay

U251MG (p53 mutated; low level of methyl guanine transferase (MGMT)) and U87MG (p53 wild type, low level of methyl guanine transferase (MGMT) cells [9] (American Type Culture Collection, Manassas, VA) were maintained at Dulbecco’s minimal essential medium (DMEM) (Thermo Fisher Scientific) with 10% Foetal Bovine Serum (FBS, Hyclone, Logan, UT) and added glutamine/pyruvate (HyClone) at 37 °C with 5% CO_2_. Cells were treated with different concentration of cisplatin/CDDP ranging from 0 to 16,6 µM (Sigma, St. Louis, MO) dissolved in Opti-MEM (Invitrogen, Carlsbad, CA) from 50 mM DMSO stock solutions (indicated in Figure Legends). After 4 h of treatment, 10% FBS was added, and cells incubated for an additional 44 h. To determine viability, PrestoBlue (Invitrogen) was added as per manufacturer’s protocol and read on a microplate reader (BioTek, Winooski, VT).

### Statistical analysis

Results are presented as mean ± s.e.m. of at least three replicates. Data sets were analyzed statistically using the JMP Statistical Discovery™ software 6.03 by SAS (Statistical Analysis Software). Statistical significance between groups was determined using Student’s t-test or one-way analysis of variance (ANOVA). Differences between the two cell lines were tested for statistical significance using the Chi Square test (Χ2). Two-tailed significance tests were performed with *p* < 0.05 considered significant. Statistical parameters for each experiment can be found within the corresponding figure legends.

## Results

### Cisplatin-based chemotherapy resistance and MEK-inhibition effects in glioblastoma

**Fig. 1:**
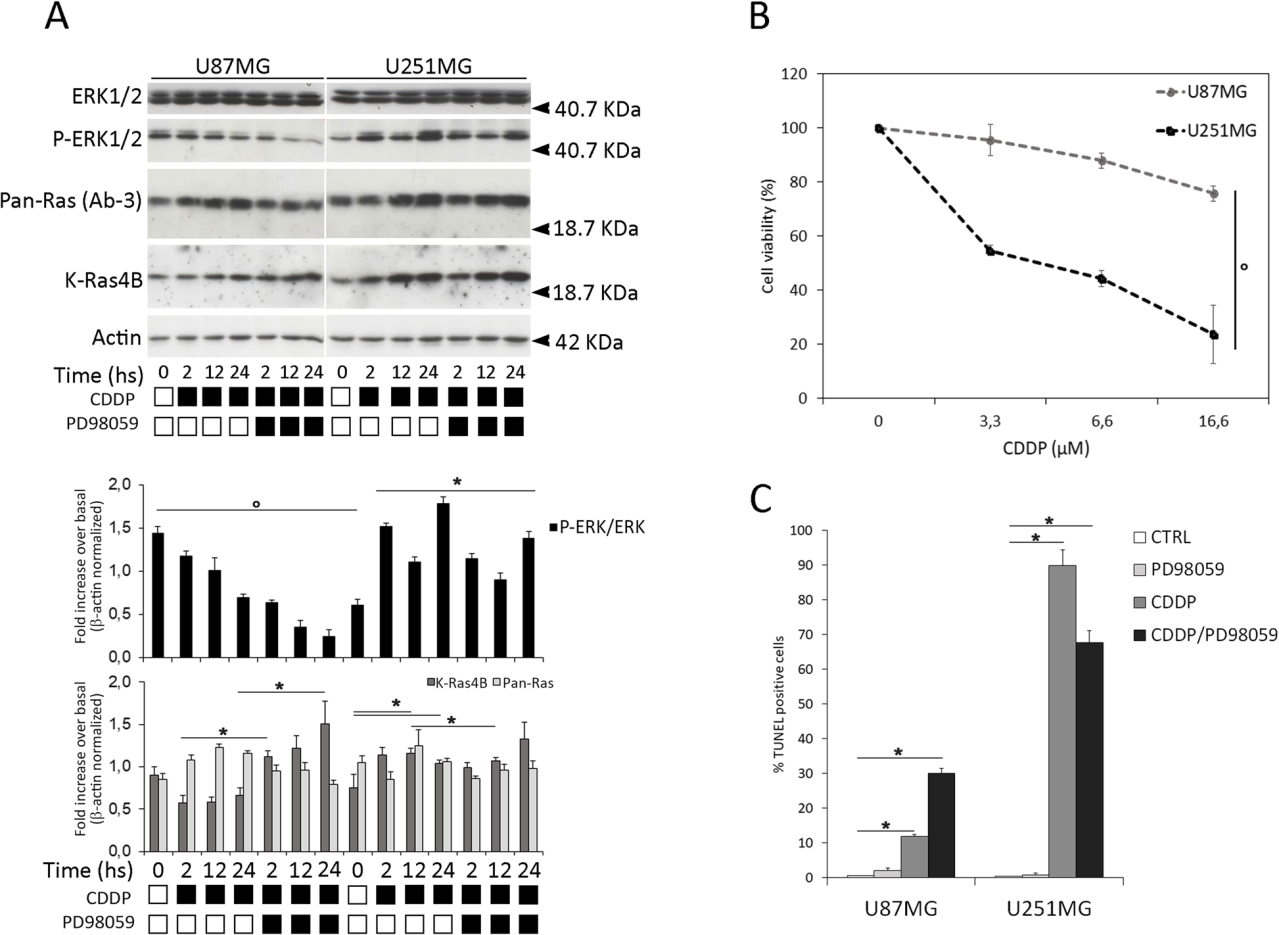
Sensitivity to cisplatin and resistance to MEK-inhibitor in glioblastoma cells. *Panel* **A**—Immunoblots showing ERK 1/2, p-ERK, pan-RAS and KRAS4B protein levels in U87MG and U251MG cells treated with cisplatin (CDDP) 16,6 µM or the MEK-inhibitor PD98059 (40 μM) for the indicated times. Western blot analysis of β-actin was performed in the same experiment, as loading control. The corresponding bar graphs show relative expression of proteins normalized to β-actin. Values are expressed as mean ± s.e.m. (*n* = 3). Differences between treatments were tested for statistical significance using Student’s matched pairs *t*-test (*P < 0.0001 compared to untreated sample) or Chi Square test (°*P* < 0.05 comparing the two cell lines). Panel **B** – Cell viability measured by MTT. Figure shows 3-(4,5-dimethylthiazol-2-yl)-2,5-diphenyltetrazolium bromide (MTT) assay at increasing doses of cisplatin (3,3 µM; 6,6 µM; 16,6 µM ( in both cultures. Cells were treated with MTT after 48 h from cisplatin (CDDP) administration and cell viability is expressed as percentage on the control (untreated cells). Panel **C** shows apoptotic cell percentage analysis assayed by TUNEL cytometry. The percentage of was evaluated after treatment with both cisplatin 16,6 µM or/and the MEK-inhibitor PD98059 (40 μM). Values are expressed as mean ± s.e.m. Differences between treatments were tested for statistical significance using Student’s matched pairs *t*-test (*P < 0.0001 compared to untreated sample) or Chi Square test (°*P* < 0.05 comparing the two cell lines)

**Fig. 2:**
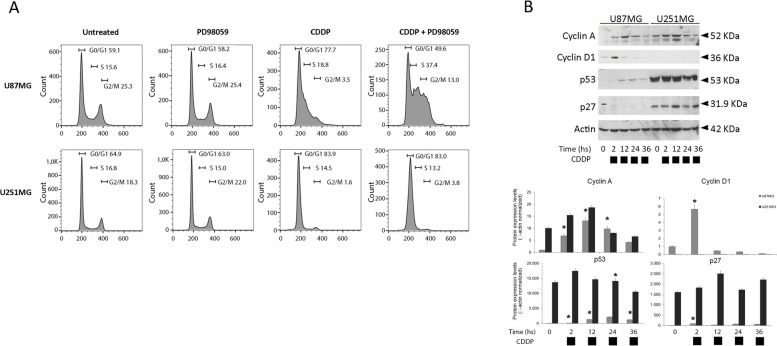
Cisplatin-based chemotherapy induces cell-cycle arrest in human glioblastoma cells. Panel **A**—Flow cytometric DNA content (propidium iodide, FL2 fluorescence) analysis in cells following FLH2 *versus* FLW2 analysis for doublet elimination. U87MG and U251MG were treated with single-dose cisplatin (CDDP) 16,6 µM and/or PD98059 (40 μM). Values are expressed as mean ± s.e.m. (*n* = 3). Differences between treatments were tested for statistical significance using Student’s matched pairs *t*-test (*P < 0.0001 compared to untreated sample). Panel **B** – Representative immunoblots showing Cyclin-A, Cyclin D1, p53 and p27 protein levels in U87MG and U251MG cells treated with cisplatin (CDDP) 16,6 µM for 2, 12, 24 and 36 h. Western blot analysis of β-actin was performed in each experiment, as loading control. Histograms below reports the relative expression of proteins normalized to β-actin

To assess the sensitivity to cisplatin of human glioblastoma, we treated in vitro two representative long-term cultures with different doses of cisplatin (CDDP) and assayed K-Ras4B protein abundance by semiquantitative Western Blot. Human U87MG and U251MG were added with cisplatin 16,6 µM for 2, 12, 24 h. Furthermore, as the activation of the MAPK signalling pathway plays an important role in GBM response to chemotherapy, we used the potent and selective non-ATP-competitive MEK1 inhibitor PD98059 (preclinical studies). Here, we measured distinct sensitivity to cisplatin of the two prototypical models of glioblastoma by using different approaches (semiquantitative Western Blot, MTT, TUNEL). Endogenous KRAS4B protein levels and ERK phosphorylation showed significant differences as measured by semiquantitative Western Blot. Cisplatin upregulates total Ras (measured by pan-Ras antibody) in both cell lines and K-Ras4B isoform (measured by isoform-specific antibody against KRAS4B) account for the total Ras amount only in U251MG (Fig. 1A and in Figure_1SBIS). Moreover, MEK-inhibitor treatment strongly de-phosphorylated ERK and contextually enhances (detected a two-fold increase) K-Ras4B protein expression in U87MG only. Inhibition of MEK by PD98059 was confirmed by de-phosphorylation of ERK1/2 in both cell lines (markedly in U251). Particularly, PD98059 alone doesn’t increase K-Ras4B expression in both cultures (Fig. 1A and Figure_1SBIS). Besides the signalling pathway in U87MG and U251MG cells, we investigated their ability to affect cell viability after 48 h of treatment by using a standardized MTT assay. Cell viability assay (MTT assay) (Fig. 1B) and cytofluorimetric analysis of fragmented DNA of apoptotic cells (TUNEL assay) (Fig. 1C) measured remarkable differences in cisplatin sensitivity among the two cultures, particularly U87MG cells were almost insensitive to CDDP. Cells were treated with different concentrations of CDDP (3,3 µM 6,6 µM 16,6 µM for 72 h and we measured a massive apoptosis only in U251MG cells (90%) versus the negligible U87MG response (10%). To determine to what extent MEK inhibition could interfere with cisplatin-induced apoptosis, these cell lines were incubated with cisplatin (CDDP) 16,6 µM and/or MEK inhibitor PD98059 40 μM for the indicated times and fluorescence-activated cell-sorting (FACS) analysis was conducted to determine the percentage of cells with a sub-G1 (apoptotic) DNA content. We observed a significantly enhanced cytotoxicity (90%) in U251MG in response to cisplatin (CDDP) (time 72 h and CDDP concentration 16,6 µM (Fig. 1 panel B), and this effect was rescued by MEK-inhibitor treatment (20%). Conversely, a percentage of 12% TUNEL-positive U87MG cells in response to chemotherapy treatment was further increased up to 30% by MEK-inhibitor (see also Supplementary Fig. 1, S1). We next used flow cytometry and Western blotting to determine whether cisplatin releases GBM cells from G_2_/M arrest and modulates G_2_/M checkpoint regulators. Cells were fixed in ice-cold ethanol and stained with propidium iodide (PI)/RNase buffer, and DNA content was analyzed by flow cytometry following FL2H *versus* FL2W analysis for doublet elimination. In the presence of different concentrations, CDDP caused a persistent accumulation of cells in G0/G1-phase without appearance of cells in G_2_/M up to 72 h in both cultures. A significant arrest in the G0/G1 cell cycle phase and subsequent decline in both S and G2/M phases were observed in U251 and, at a lesser extent in U87MG cells following cisplatin treatment (72 h at 5 µM concentration) (Fig. 2A). Notably, the percentage of U87MG cells in the G1 remains almost constant, except for co-treatment with MEK-inhibitor. S and G2 phases (and the level of expression of cyclin D1 in panel B) decreased in U87MG cells (Fig. 2A). Consistent with these results, upregulation of cyclin D1 expression (and cyclin A) was detected in U87MG cells treated with cisplatin, but not in cisplatin treated U251MG cells. Interestingly, evaluation of cyclin D1 expression in thigh confluence was higher than lower density. The abnormal expression of cyclin D1 at a high cell density was observed in both conditions, growing and starvation, and in cisplatin-treated cells over the time-course (Supplementary Fig. 2, Figure_S2). The levels of the cell cycle inhibitor p27, but not cyclin D1, were dramatically increased in U251MG cells, suggesting that p53-mutated glioblastoma may be more sensitive to cisplatin-induced apoptosis. Interestingly, we observed absence of expression of cyclin D1 and p27 in U251MG (Fig. 2B).

### Cisplatin-based chemotherapy resistance is defined by HVR K-RAS post-translational modification in human glioblastomas

**Fig. 3:**
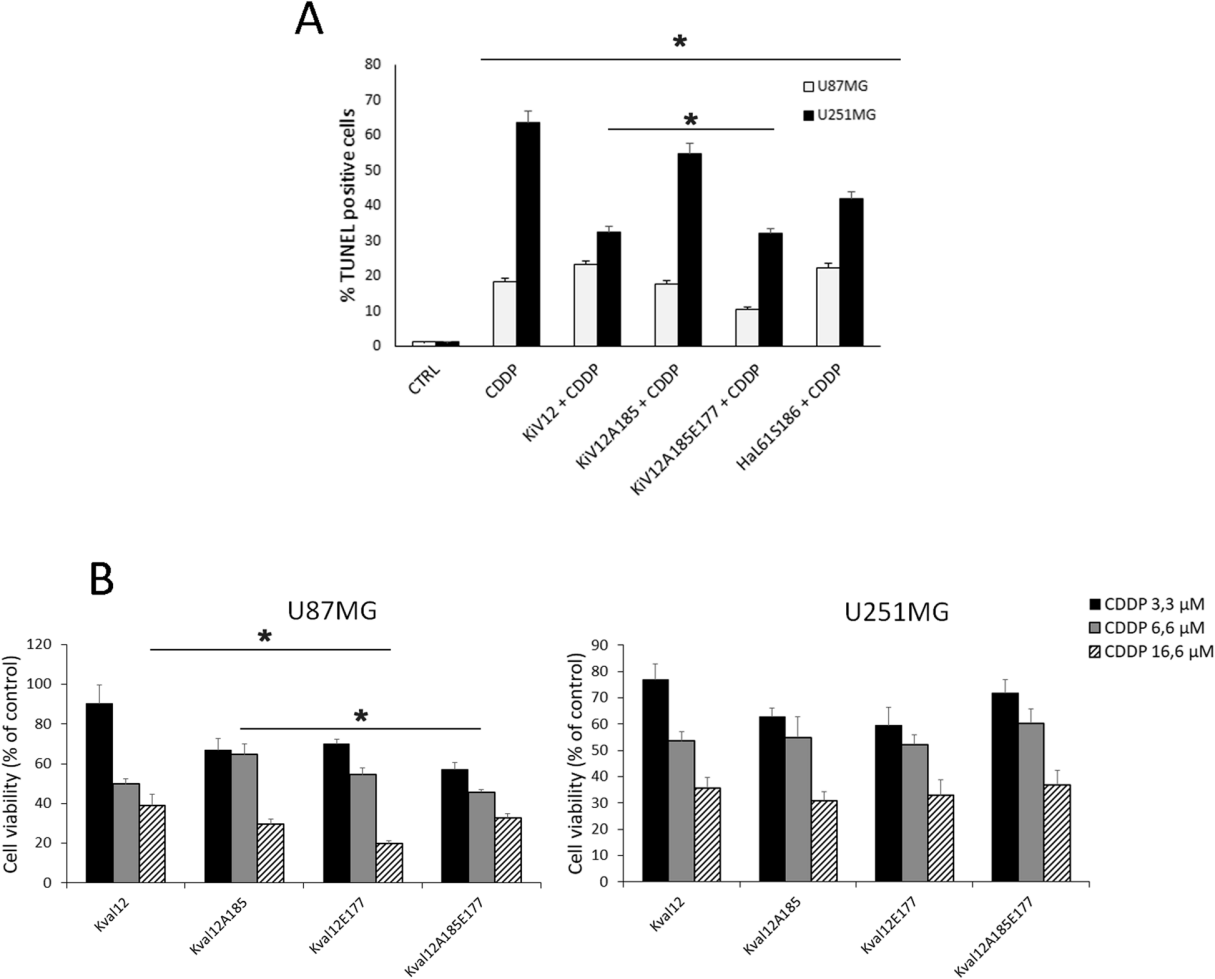
Sensitivity to cisplatin in KRAS HVR-mutants. Panel **A**—Cisplatin-induced apoptosis assayed by (Terminal deoxynucleotidyl transferase dUTP nick end labeling) TUNEL is shown in cells overexpressing plasmids coding for oncogenic KRAS carboxyl-terminal hypervariable region (HVR)-mutants KRAS-G12V, KRAS-G12V-C185A, KRAS-G12V-C185A-K177E and HRASL61S186. Histogram shows apoptotic cell percentage analysis assayed by TUNEL. Panel **B** 3-(4,5-dimethylthiazol-2-yl)-2,5-diphenyltetrazolium bromide (MTT) assay at increasing doses of cisplatin (3,3 µM; 6,6 µM; 16,6 µM in U87MG and U251MG. The cells were treated with MTT after 48 h from cisplatin (CDDP) administration and cell viability is expressed as percentage on the control (untreated cells). Values are expressed as mean ± s.e.m. Differences between treatments were tested for statistical significance using Student’s matched pairs *t*-test (*P < 0.0001 compared to untreated sample)

Experiments of gain-of-function were performed by overexpressing plasmids coding for oncogenic KRAS carboxyl-terminal hypervariable region (HVR)-mutants. We measured apoptosis (TUNEL Panel A) and cell viability (MTT Panel B) in response to increasing doses of cisplatin in these cells over-expressing oncogenic KRAS^G12V^ (Fig. 3). Over-expression of KRAS^G12V^, KRAS^G12VC185A^, KRAS^G12VC185AK177E^ mutants was obtained by transient transfection in both U87MG and U251MG cells and assayed at 72 h (see Methods and Figure S3). Interestingly, overexpression of the constitutively active mutant KRAS^G12V^ induced opposite response to CDDP treatment. Percent cell apoptotic cells were assessed for both cell lines that were either treated or untreated with CDDP. We measured increased values of TUNEL-positive U87MG over-expressing KRAS^G12V^ cells (23,2% vs 18,4%) compared to decreased values (60% vs 30%) in U251 in response to CDDP treatment. Moreover, the mutant KRAS^G12VC185A^, in which is prevented the farnesylation of the residue 185, do not alter the response to CDDP; on the other hand, the triple mutant KRAS^G12VC185AK177E^ (in which the polybasic region of K-RAS HVR is partially neutralized) exerts a protective role in cisplatin chemosensitivity in both (Fig. 3A). Next, we measured the cell viability of transiently transfected KRAS^G12V^ mutants by using a standardized MTT assay (Fig. 3B). We detected only slightly differences between KRAS^G12V^ mutants on viability measured by mitochondrial activity. This effect could be mediated by increased intracellular and mitochondrial reactive oxygen species and decreased mitochondrial membrane potential (ΔΨm) induced by cisplatin [].

### KRAS expression in human glioblastomas

Primary human glioblastomas show high mRNA expression of KRAS compared to long-term cultures (Supplementary Fig. 4, Figure_S4). The KRAS expression was evaluated by real-time polymerase chain reaction (RT-PCR) in a panel of traditional xenograft cell lines and patient-derived xenograft models. We analyzed a panel of glioblastoma samples, spanning from primary cultures (GBM, WHO grade 4, see methods) to several human long-term cultures U87MG, U87MG^IDH1mut^, U251MG, T98G, SW1783, and Ln229 and T98G. Normal Human Astrocytes (NHA), Neural Stem Cells (SC30) and the human fetal glial cell line SVGp12 *KRAS* mRNA abundance were used as references samples. We detected *KRAS* transcripts are highly abundant in all primary samples (GBM#C, GBM#D, GBM#F and GBM#M) compared to classical long-term cell lines. It’s interesting to note that fetal glial cell line SVGp12 shows relatively high level of *KRAS* mRNA, accordingly with patterns of Ras expression in mouse brain (cerebral cortex) during the development [49]. Human long-term cultures (U87MG w.t. and mutant IDH1, U251MG and Ln229 showed comparable abundance of mRNA transcript (Supplementary Fig. 4, Figure_S4). Relative high protein abundance of endogenous K-Ras4B in long-term cultures and primary cultures (GBM, WHO grade 4) were assessed by semiquantitative western blot analysis with a panel of commercial antibodies specific for KRAS4B [50] (data not shown).

## Discussion

In the current study, we examined the *KRAS* gene involvement in platinum-based chemotherapy in human glioblastoma. Our results revealed high *KRAS* expression in primary human GBM tumors compared to several GBM cell lines. An in vitro examination of cell viability, cell cycle progression and apoptosis in two glioblastoma cell lines, U87MG and U251MG were used to study cisplatin responsiveness. Firstly, we examined endogenous KRAS expression in response to cisplatin, studying the involvement of the effector pathway RAF/MEK/ERK mitogen-activated protein kinase (MAPK) cascade. Then, including gain-of-function experiments with plasmids coding for oncogenic KRAS^G12V^ carboxyl-terminal hypervariable region (HVR)-mutants, we examined the responsiveness to oncogenic over-expression.

The antitumor efficacy of cisplatin is unquestionable. Platinum-based chemotherapy remains popular for treating cancers, especially in patients with genetic or pathological profiles that respond poorly to targeted therapies. Although cisplatin is used for adjuvant chemotherapy against glioma [51] and therein references), intrinsic and acquired resistance restricts cisplatin application. Preclinical in vitro data reported CDDP half maximal inhibitory concentrations (IC_50_) in glioblastoma considerably lower (hundred times) than that temozolomide (TMZ) in cell lines commonly used for research on gliomas The former exhibited strong resistance to cisplatin in our experiments, which agreed with its performance in xenograft transplantation models [15]. The median in vitro IC_50_ of cisplatin was 8 μM which is consistent with previous in vitro tests in glioblastoma and other tumour cells [52, 53]. Viability, apoptosis, and cell cycle assays showed remarkable differences, especially in terms of massive (90%) *versus* negligible (12%) apoptotic response in U251MG and U87MG respectively. These differences may be partially due to their different genetic background involving their mutational status [39]. The molecular signature of glioblastoma (proneural, classical and mesenchymal GBM) with distinctly different patterns of gene expression is, regrettably, poorly representated by cell line models that are still widely used. These two cultures differ mainly in their genetic background respect p53 status that affect cell cycle progression during cisplatin administration. Particularly, p53 has opposing effects in gliomas treated with methylating agents and, therefore, the p53 status should be considered when deciding which therapeutic drug to use [4].

Dysregulated signalling represents an important conserved oncogenic mechanism. The dysfunctional signalling in tumours arises also by rewiring of signalling pathways, also determining the response to treatment. KRAS signalling necessarily relies on ERK/MEK signalling and, MEK-inhibitors as single agent or in combinatorial setting are at the leading-edge treatment for many cancers, including glioblastoma [19]. Actually, clinically approved MEK inhibitors (i.e.Trametinib) showed no apparent benefit of blocking MEK [35]. We therefore sought to investigate this further as a potential explanation for MEK-resistance in glioblastoma. By using the MEK-inhibitor PD98059, we observed an increased KRAS protein expression and the concomitant ERK dephosphorylation exerting opposite effect on percentage of apoptotic cells and, doing so, blocked the progression at various stages of the cell cycle. The MEK/ERK pathway is considered to enhance survival and confer resistance against radio- and chemotherapy. Blocking MEK signaling in GBM is clearly antiproliferative and, the absence of MEK activity, did not cause cell death per se but sensitize cells for apoptosis induced by chemotherapy. Moreover, as reported by others [54], inhibition of the PI3K but not the MEK/ERK pathway sensitizes human glioma cells to alkylating drugs these are not investigated in our experiments.

Since Cyclin D1 is a major regulator of cell cycle progression, we therefore sought to investigate cyclins modulation in our experiments. Cyclins A, E and D1 and p27 expression were assayed by immunoblot analysis over time exposure of 36 h. Our results do not confirm previous observations showing cyclin D1 expression in U251MG cultures [55], instead U87MG p53 wild type only shows cyclin D1 induction as marker of diminished cell cycle arrest. Moreover, it is well known that p53 affects both the duration of G2/M arrest and the fate of alkylating-treated human glioblastoma cells [16]. Here, MEK inhibitor was capable of mitigating cisplatin-induced G2/M arrest in U87MG (p53 wild-type), as evidenced by the reduction in cell accumulation in the G2/M phase of cell cycle following cisplatin treatment.

The importance of mutations in RAS oncogenes in tumorigenesis, cancer progression and resistance to treatment has been demonstrated in numerous model systems in vitro and in vivo. The majority of KRAS are localized in codon 12 (changing glycine to either valine, aspartic acid, or arginine) involving in a constitutive and aberrant activation of the downstream KRAS signaling cascade. It’s well known that constitutively activated KRAS^G12D^ is not sufficient for astrocytoma initiation but rather is required for progression to high-grade tumors [36]. Here, we reported experiments of gain-of-function obtained by over-expressing oncogenic KRAS^G12V^ in glioblastoma, studying the cisplatin resistance in relationship with single-point-mutations HVR K-RAS post-translational modification. Transient over-expression of mutants KRAS^G12V^, KRAS^G12VC185A^, KRAS^G12VC185AK177E^ induced different response to cisplatin treatment depending on the tumour’s context. Meanwhile oncogenic KRAS^G12V^ was able to rescue cisplatin-induced apoptosis, otherwise the mutant KRAS^G12VC185A^, in which is prevented the farnesylation of the residue 185, partially rescued this response. Of note, the triple mutant KRAS^G12VC185AK177E^ (in which the polybasic region of K-RAS HVR is partially neutralized) mimicked the oncogenic KRAS^G12V^ response as well as Harvey mutant HRAS^L61S186^. From these results, we conclude that, at least in U251MG glioblastoma cultures, the overexpression of an oncogenic KRAS mutations modulate chemoresistance in vitro, not necessarily coupled with effect on proliferation/viability. Its worthy of note that oncogenic KRAS^G12D^ or KRAS^G12C^ are involved in the generation of intracellular reactive oxygen species [56] as well as cisplatin is involved in oxidative metabolism accompanied the cisplatin-induced inhibition of cancer cell growth in vitro and in vivo [57]. Our current data define a novel role of *KRAS* in GBM and elucidate a molecular mechanism underlying KRAS-mediated GBM chemoresistance. Particularly, chemo treatment with cisplatin induces viability and apoptotic changes in glioblastoma cells in vitro, and KRAS proteins can reprogram cell state when ectopically expressed. This provides further insights into cisplatin responsiveness of glioblastoma cancer which could ultimately lead to clinical opportunities to manipulate KRAS pathways/activity to maximize patient benefit.

### Supplementary Information

**Additional file 1.**

**Additional file 2.**

**Additional file 3.**

**Additional file 4.**

**Additional file 5.**

**Additional file 6.**

## Data Availability

All data generated or analyzed during this study are included in this published article [and its supplementary information files].

## Abbreviations

KRAS: Kirsten Ras human gene
GBM: Glioblastoma
CDDP: Cisplatin
MAPK: Mitogen Activated Protein Kinase
RT-PCR: Reverse-transcription polymerase chain reaction
WB: Western Blot
ERK: Extracellular signal-regulated kinase
MEK: MAP-ERK kinase
HVR: Hyper Variable Region
TMZ: Temozolomide
HVR: Carboxyl-terminal hypervariable region
MGMT: O6-methylguanine-DNA-methyltransferase
PI: Propidium iodide
MTT: 3-(4,5-Dimethylthiazol-2-yl)-2,5-diphenyltetrazolium bromide
TUNEL: Terminal deoxynucleotidyl transferase dUTP nick end labeling
