# Supplementary material for: KRAS is a molecular determinant of platinum responsiveness in glioblastoma
Source: BMC Cancer. 2024 Jan 15;24:77. doi: 10.1186/s12885-023-11758-6 (PMC10789061; doi:10.1186/s12885-023-11758-6)
Supplement: Supplementary file 1 — Additional file 1. [file 12885_2023_11758_MOESM1_ESM.docx]

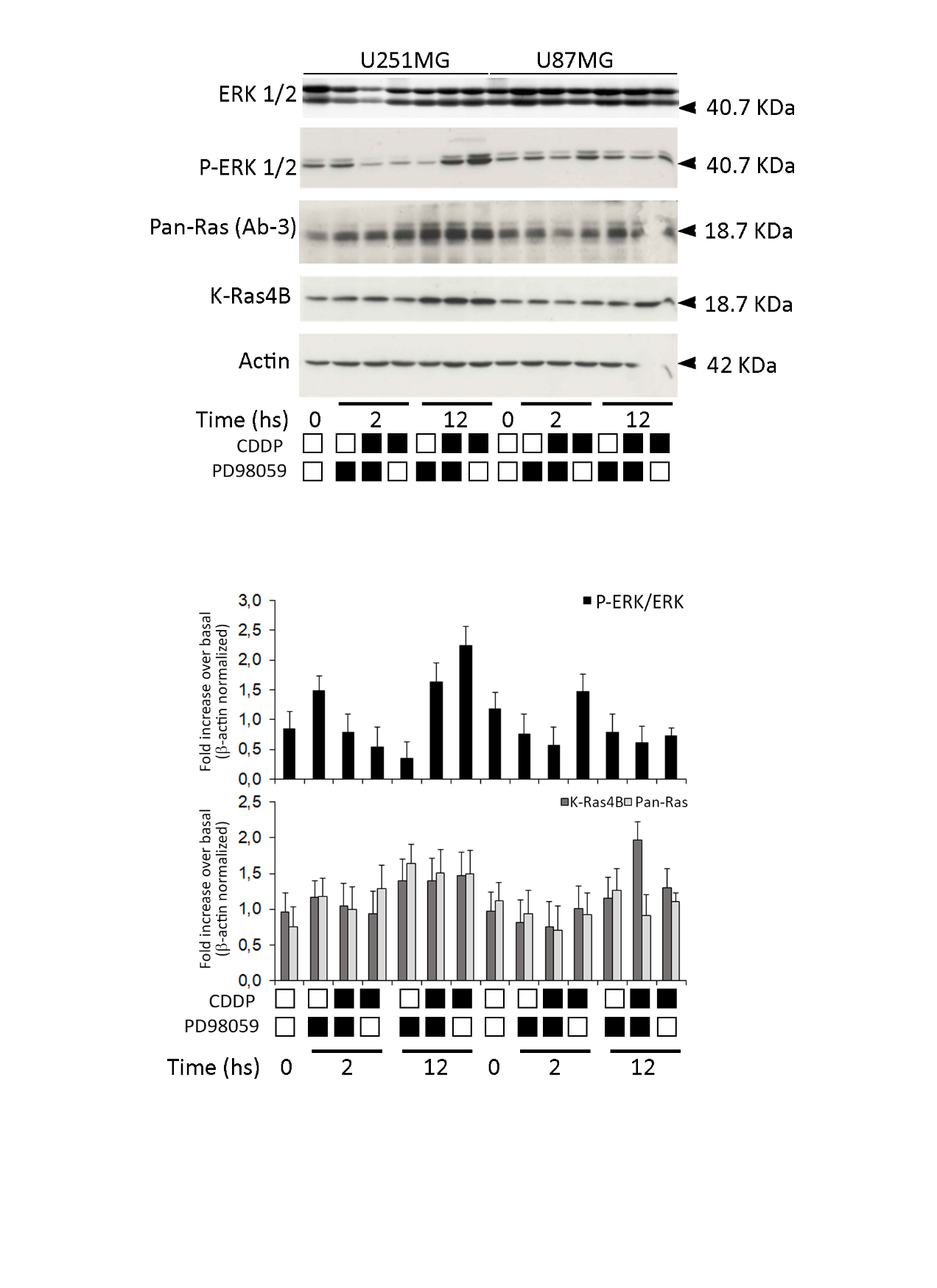


**Figure 1 bis - Sensitivity to cisplatin and resistance to MEK-inhibitor in glioblastoma cells.**  Immunoblots showing ERK 1/2, p-ERK, pan-RAS and KRAS4B protein levels in U87MG and U251MG cells treated with cisplatin (CDDP) 16,6 µM or the MEK-inhibitor PD98059 (40 μM) for the indicated times. Western blot analysis of β-actin was performed in the same experiment, as loading control. The corresponding bar graphs show relative expression of proteins normalized to β-actin.  Values are expressed as mean ± s.e.m. (𝑛 = 3).
