# Supplementary material for: KRAS is a molecular determinant of platinum responsiveness in glioblastoma
Source: BMC Cancer. 2024 Jan 15;24:77. doi: 10.1186/s12885-023-11758-6 (PMC10789061; doi:10.1186/s12885-023-11758-6)
Supplement: Supplementary file 2 — Additional file 2. [file 12885_2023_11758_MOESM2_ESM.docx]

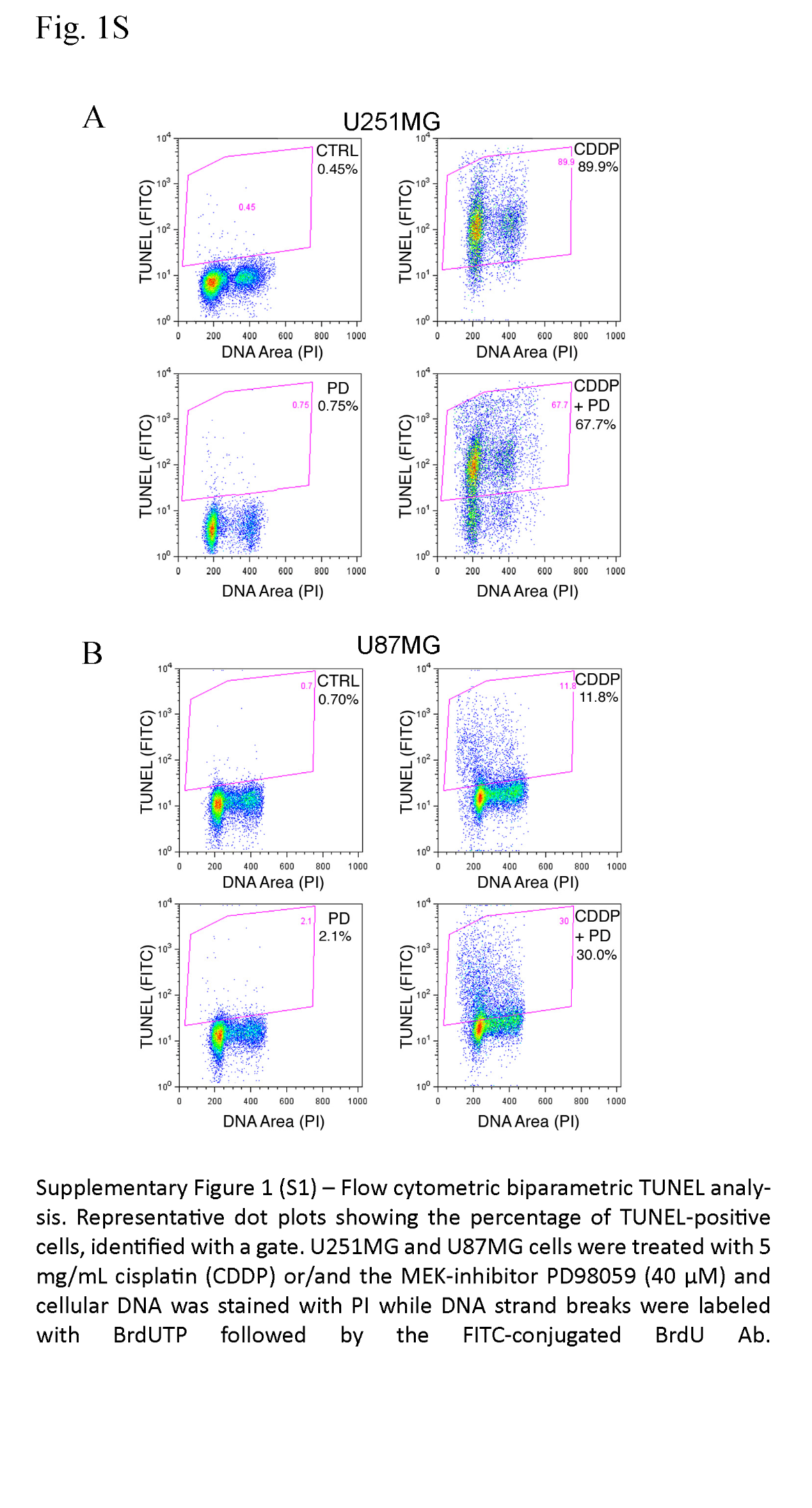


**Supplementary Figure 1 (S1) – Flow cytometric biparametric TUNEL analysis.** Representative dot plots showing the percentage of TUNEL-positive cells, identified with a gate. U251MG and U87MG cells were treated with 16,6 µM ~~5 mg/mL~~ cisplatin (CDDP) or/and the MEK-inhibitor PD98059 (40 μM) and cellular DNA was stained with PI while DNA strand breaks were labeled with BrdUTP followed by the FITC-conjugated BrdU Ab.
