# Supplementary material for: KRAS is a molecular determinant of platinum responsiveness in glioblastoma
Source: BMC Cancer. 2024 Jan 15;24:77. doi: 10.1186/s12885-023-11758-6 (PMC10789061; doi:10.1186/s12885-023-11758-6)
Supplement: Supplementary file 3 — Additional file 3. [file 12885_2023_11758_MOESM3_ESM.docx]

Fig. 2S


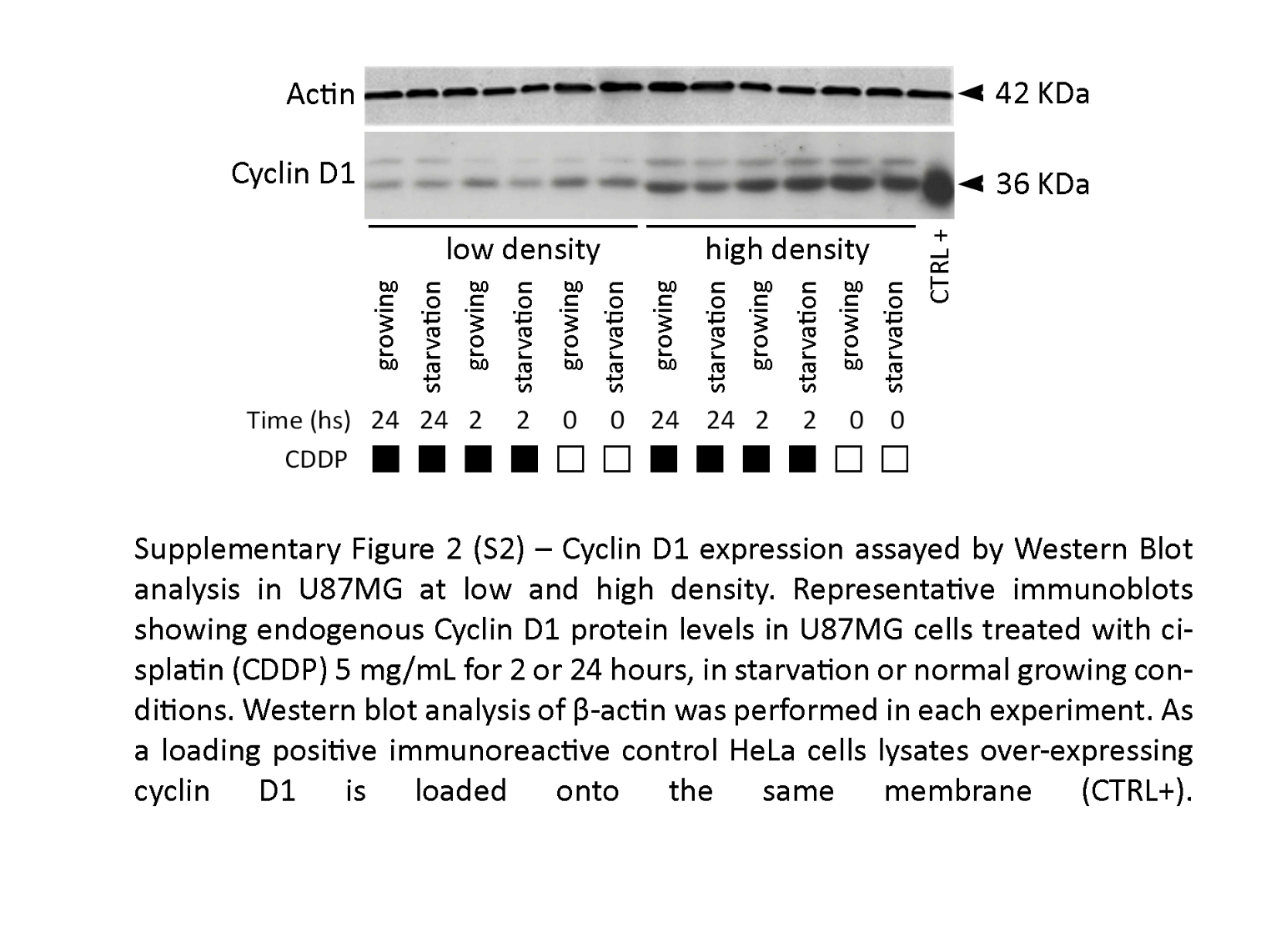


***Supplementary Figure 2 (S2)*** **– Cyclin D1 expression assayed by Western Blot analysis in U87MG at low and high density.**Representative immunoblots showing endogenous Cyclin D1 protein levels in U87MG cells treated with cisplatin (CDDP) 16,6 µM for 2 or 24 hours, in starvation or normal growing conditions. Western blot analysis of β-actin was performed in each experiment. As a loading positive immunoreactive control HeLa cells lysates over-expressing cyclin D1 is loaded onto the same membrane (CTRL+).
