# Supplementary material for: KRAS is a molecular determinant of platinum responsiveness in glioblastoma
Source: BMC Cancer. 2024 Jan 15;24:77. doi: 10.1186/s12885-023-11758-6 (PMC10789061; doi:10.1186/s12885-023-11758-6)
Supplement: Supplementary file 4 — Additional file 4. [file 12885_2023_11758_MOESM4_ESM.docx]

Fig. 3S


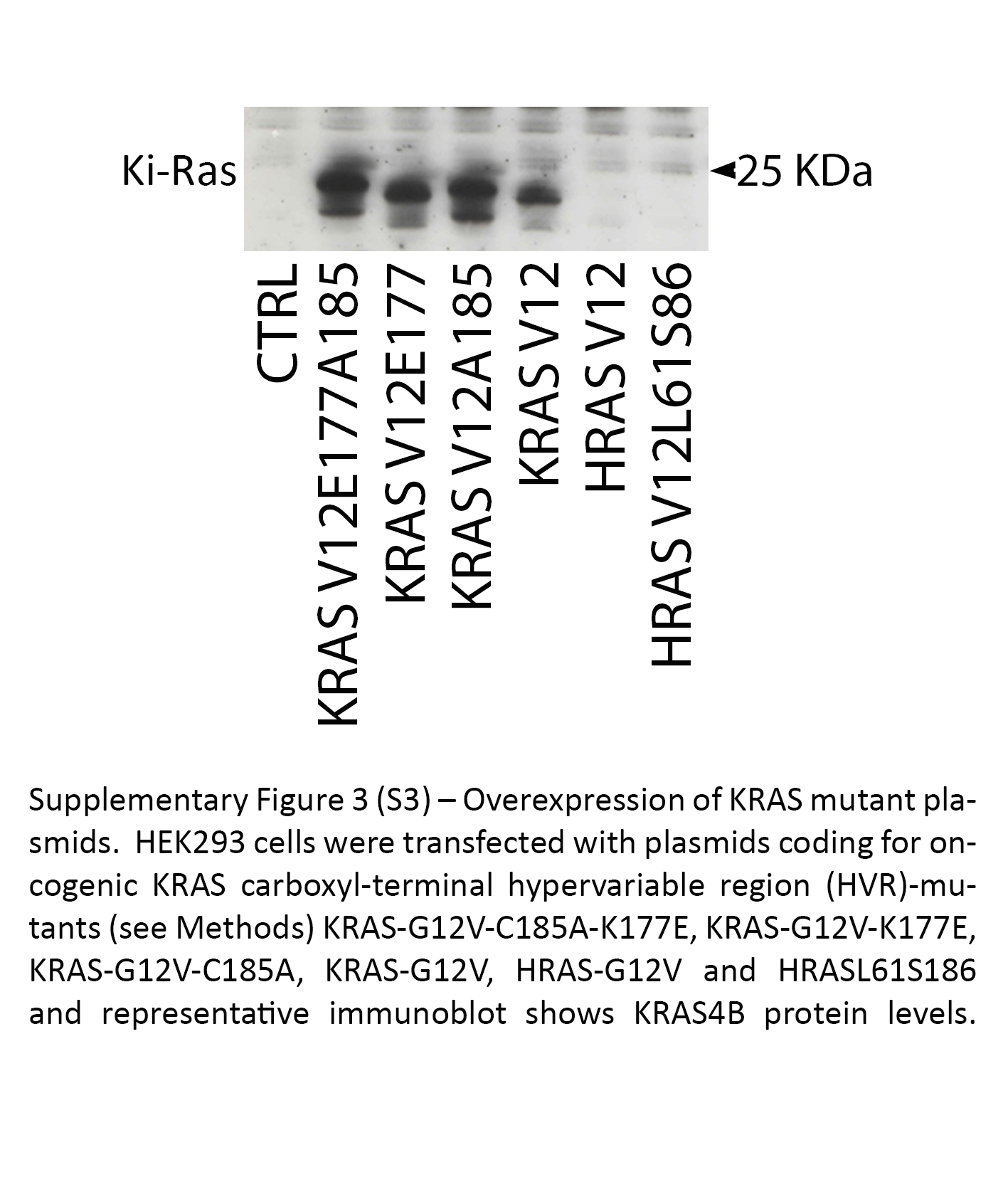


***Supplementary Figure 3 (S3)*** – **Overexpression of K-RAS4B mutant plasmids.**  HEK293 cells were transfected with plasmids coding for oncogenic K-RAS4B carboxyl-terminal hypervariable region (HVR)-mutants (see Methods) KRAS-G12V-C185A-K177E, KRAS-G12V-K177E, KRAS-G12V-C185A, KRAS-G12V, HRAS-G12V and HRASL61S186 and representative immunoblot shows KRAS4B protein levels.
