# Supplementary material for: KRAS is a molecular determinant of platinum responsiveness in glioblastoma
Source: BMC Cancer. 2024 Jan 15;24:77. doi: 10.1186/s12885-023-11758-6 (PMC10789061; doi:10.1186/s12885-023-11758-6)
Supplement: Supplementary file 5 — Additional file 5. [file 12885_2023_11758_MOESM5_ESM.docx]

Fig. 4S


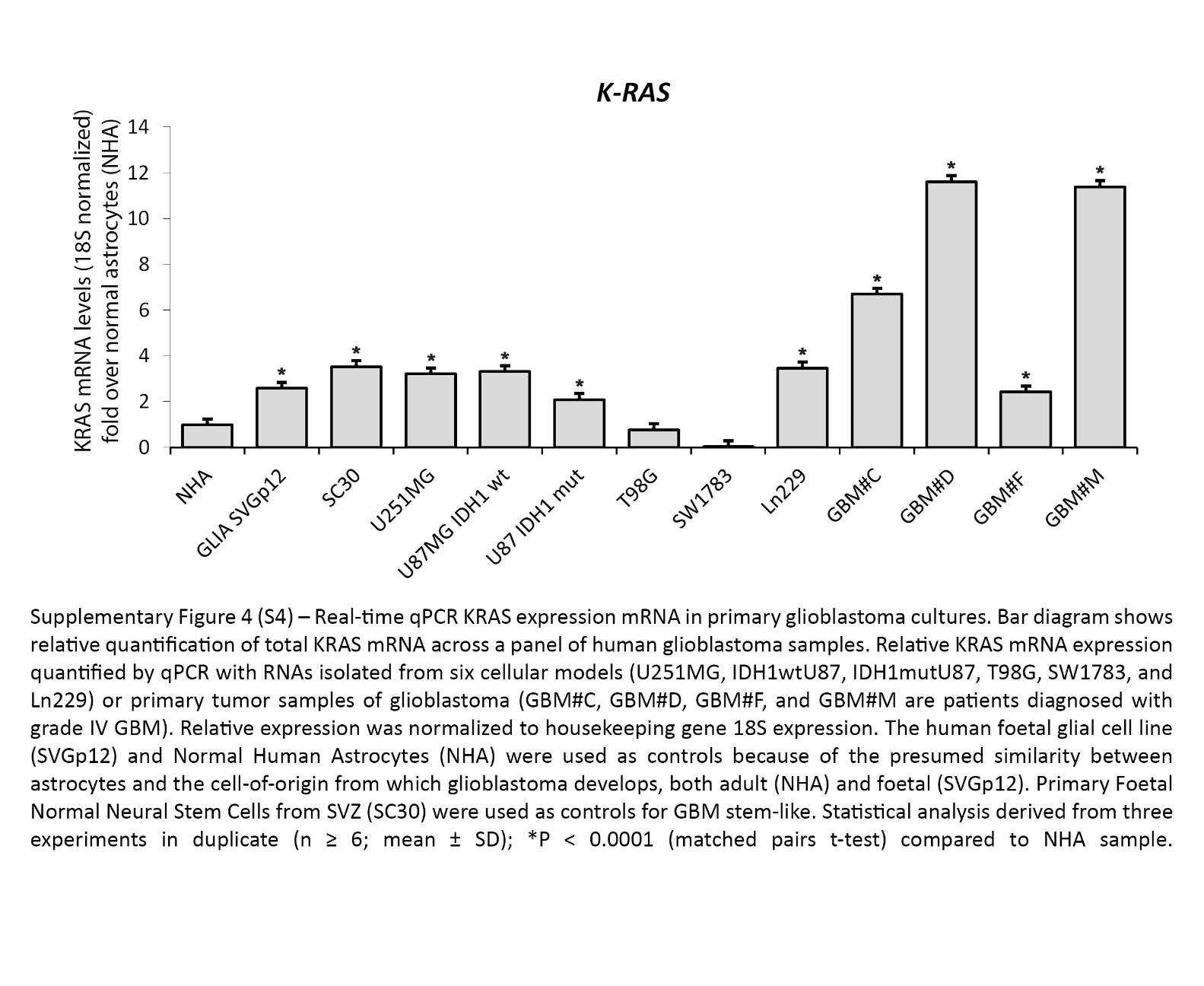


***Supplementary Figure 4 (S4) – Real-time qPCR K-RAS4B expression mRNA in primary glioblastoma cultures.*** Bar diagram shows relative quantification of total KRAS mRNA across a panel of human glioblastoma samples. Relative K-RAS4B mRNA expression quantified by qPCR with RNAs isolated from six cellular models (U251MG, IDH1^wt^U87, IDH1^mut^U87, T98G, SW1783, and Ln229) or primary tumor samples of glioblastoma (GBM#C, GBM#D, GBM#F, and GBM#M are patients diagnosed with grade IV GBM). Relative expression was normalized to housekeeping gene 18S expression. The human foetal glial cell line (SVGp12) and Normal Human Astrocytes (NHA) were used as controls because of the presumed similarity between astrocytes and the cell-of-origin from which glioblastoma develops, both adult (NHA) and foetal (SVGp12). Primary Foetal Normal Neural Stem Cells from SVZ (SC30) were used as controls for GBM stem-like. Statistical analysis derived from three experiments in duplicate (n ≥ 6; mean ± SD); *P < 0.0001 (matched pairs t-test) compared to NHA sample.
