# Supplementary material for: KRAS is a molecular determinant of platinum responsiveness in glioblastoma
Source: BMC Cancer. 2024 Jan 15;24:77. doi: 10.1186/s12885-023-11758-6 (PMC10789061; doi:10.1186/s12885-023-11758-6)
Supplement: Supplementary file 6 — Additional file 6. [file 12885_2023_11758_MOESM6_ESM.docx]

***Supplementary Figure S5***


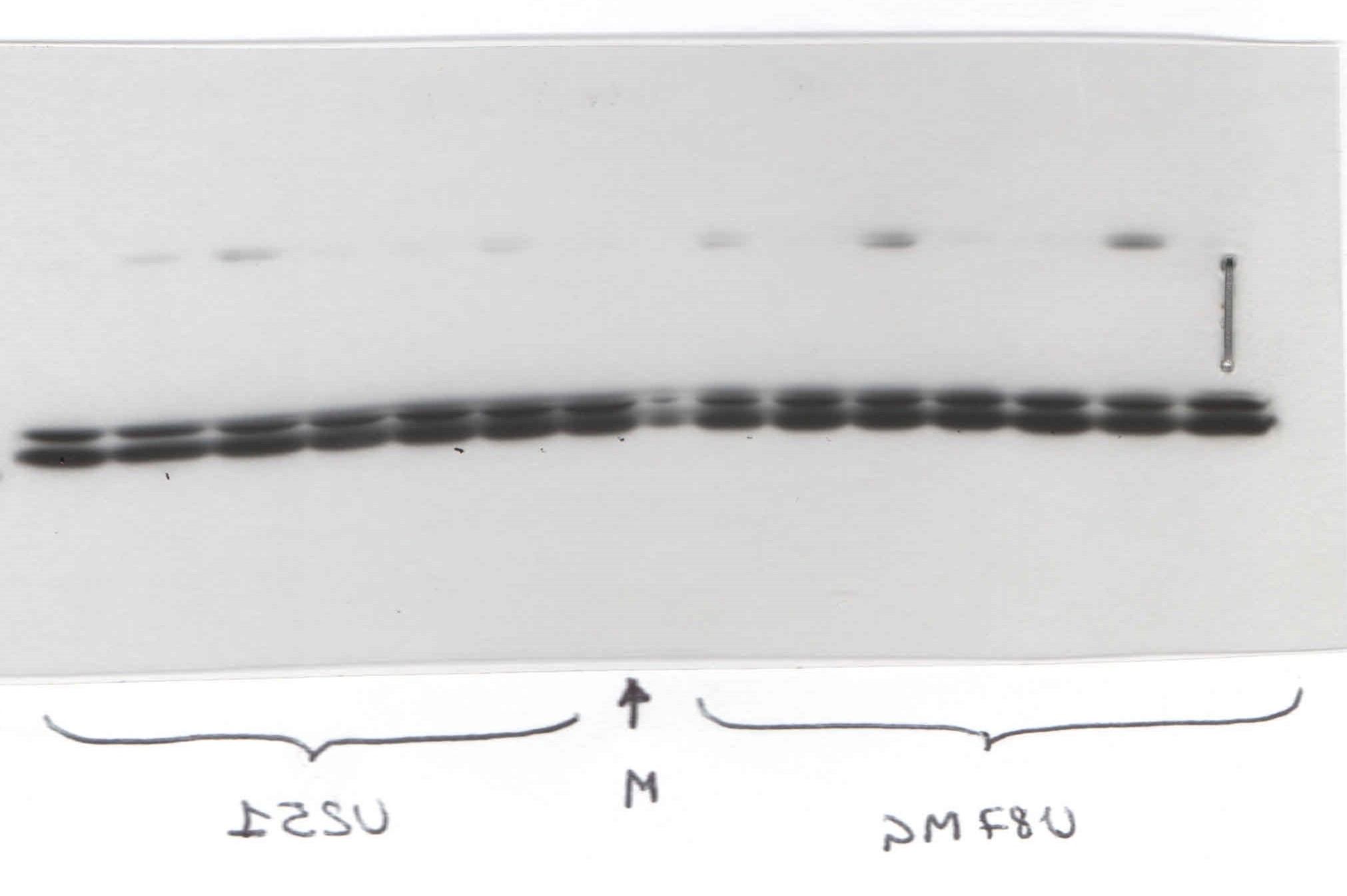
ERK 1/2


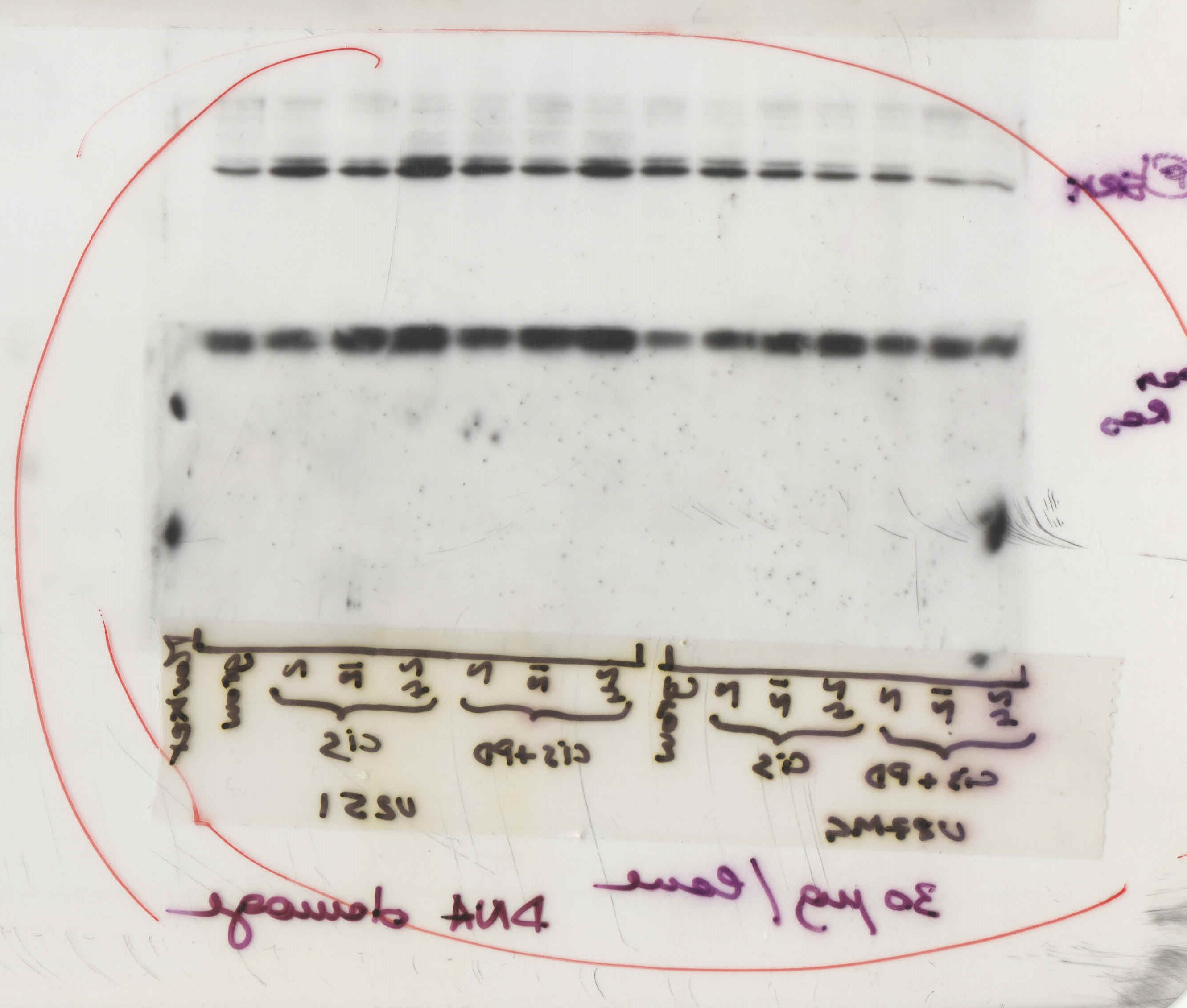
 P-ERK 1/2 and pan-Ras


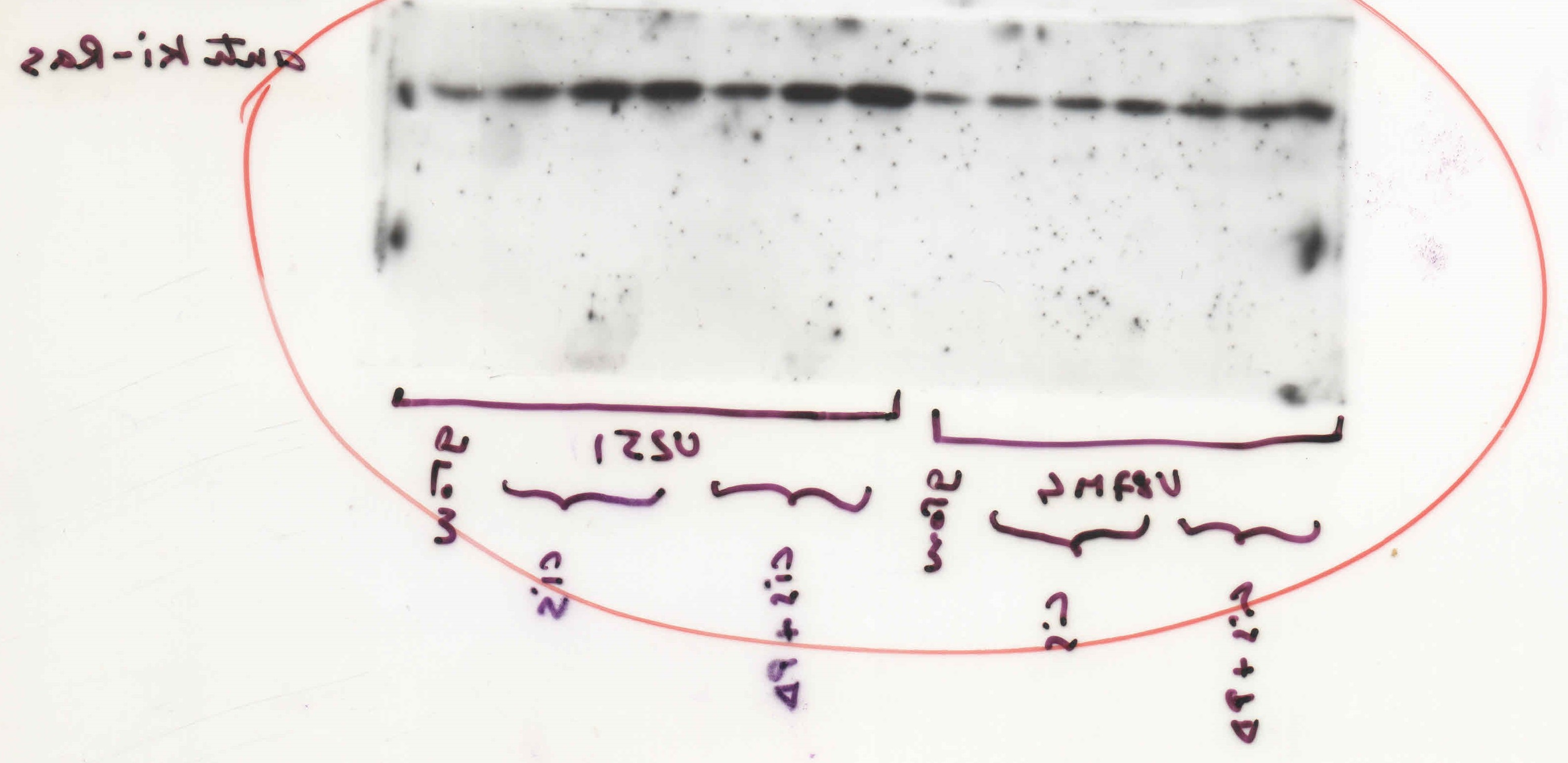
 K-Ras4B


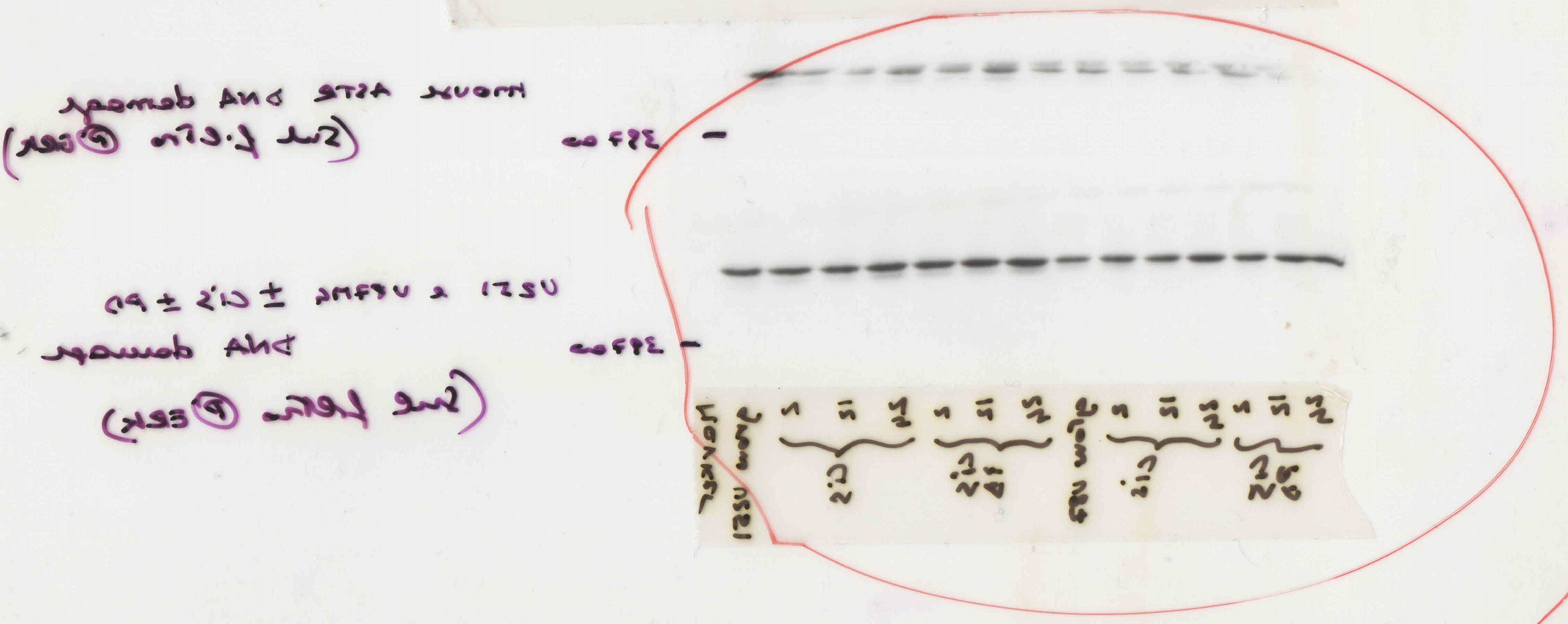
 β-actin

***Figure S5. Uncropped versions of the western blot used in this manuscript (corresponding to Figure 1 panel A).*** Original gels of Western Blot analysis of Figure 2 panel B: immune-reactive bands corresponding to specific antibodies against ERK 1/2, P-ERK 1/2, Pan-Ras, K-Ras4B and β-actin.

***Supplementary Figure S6***


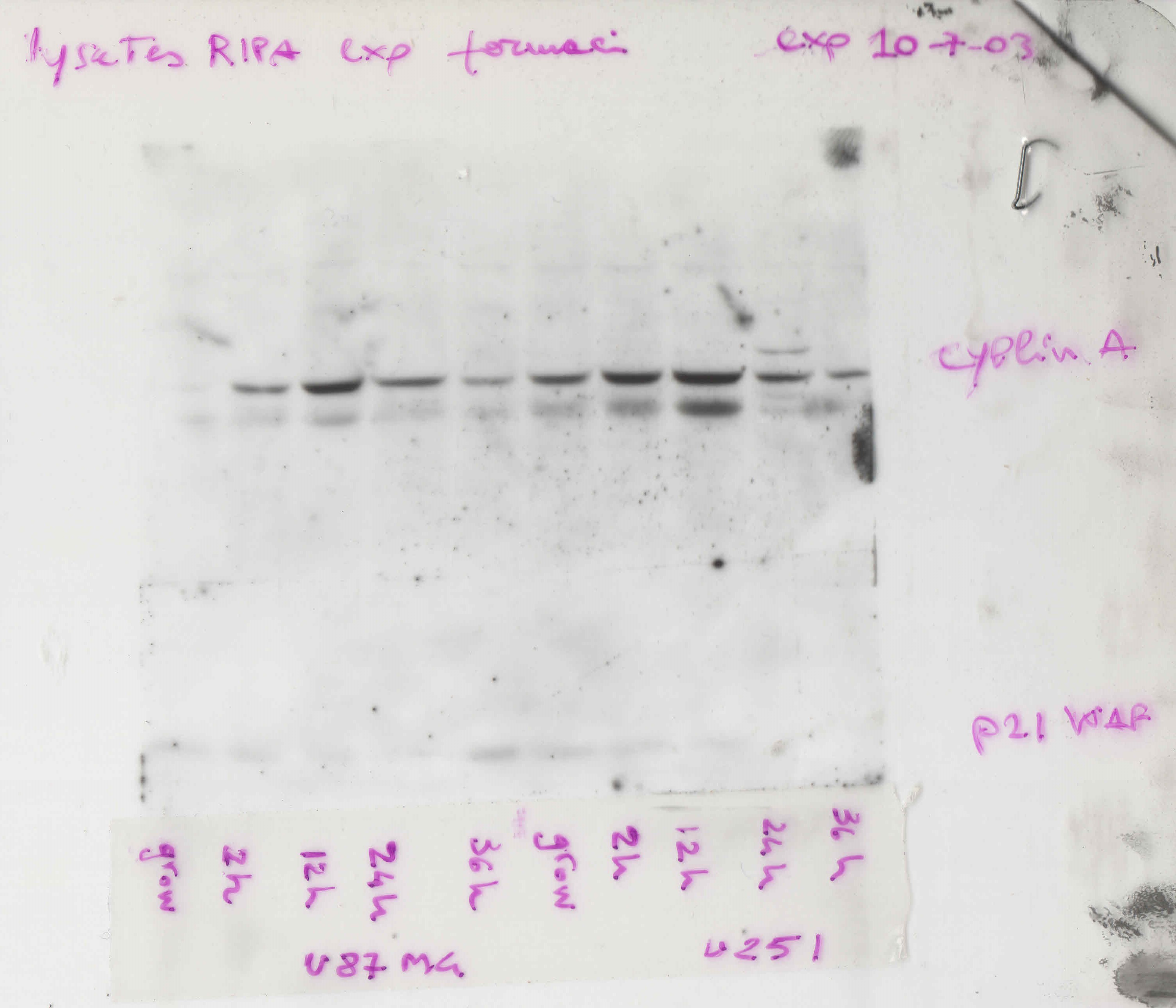
 Cyclin A


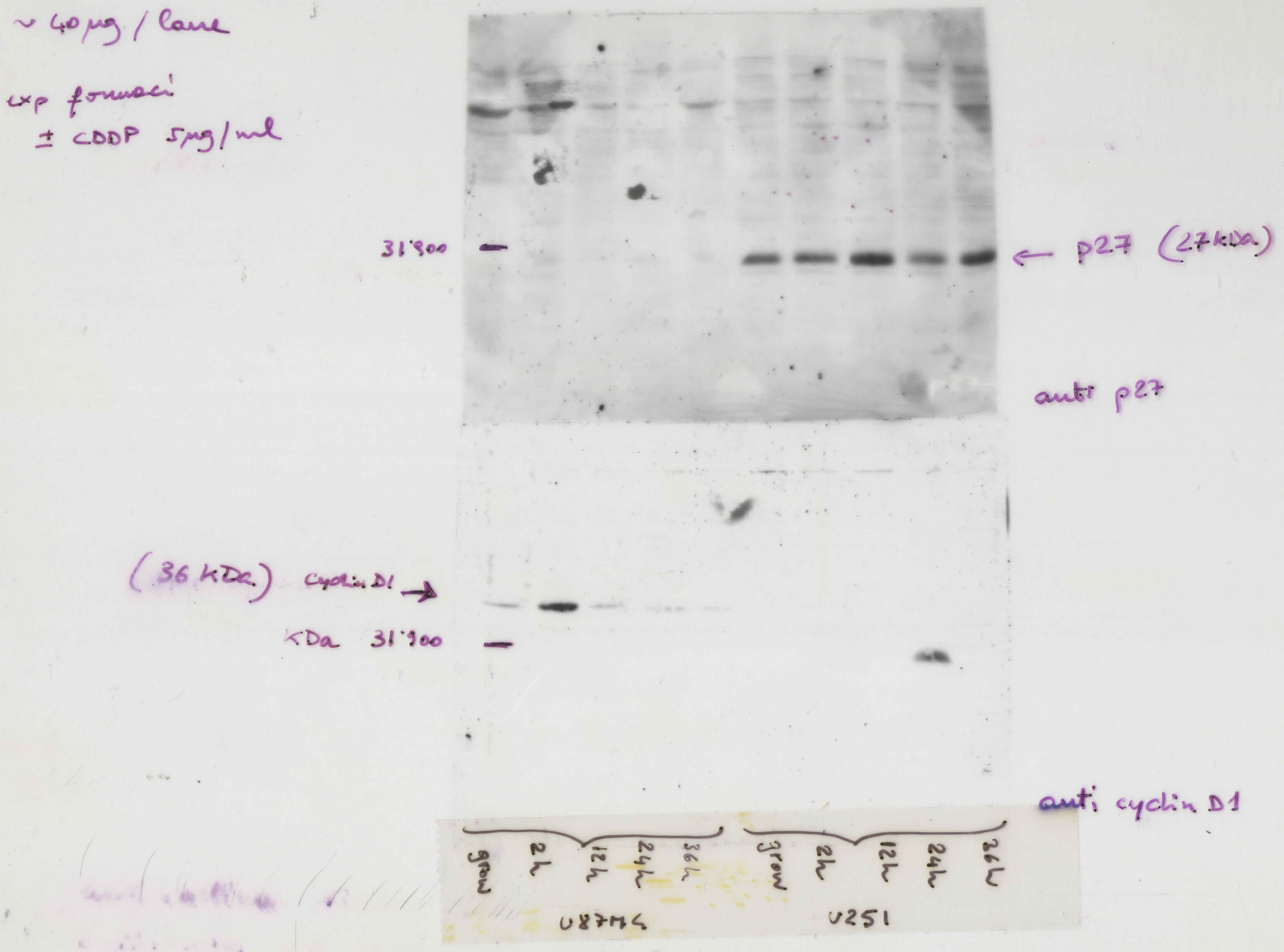
p27 and Cyclin D1


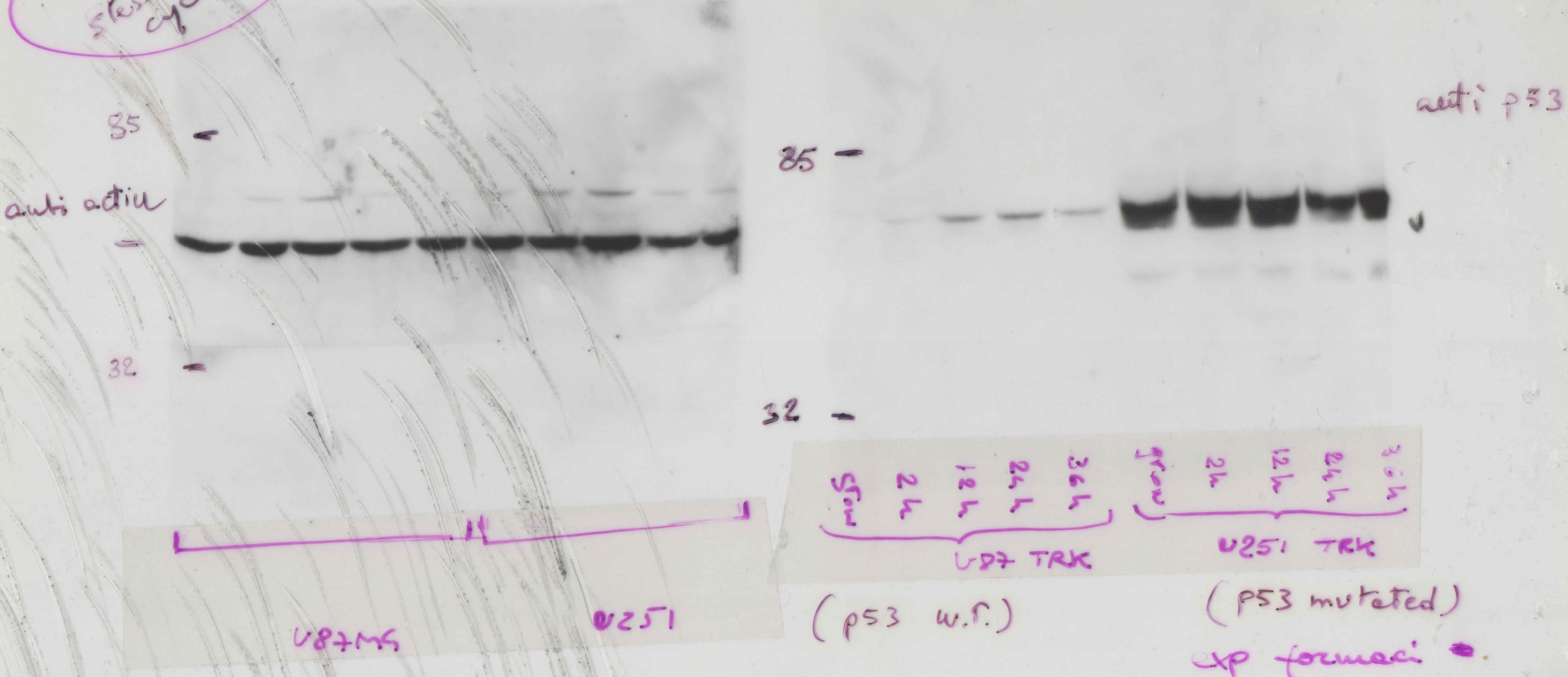
p53 and β-actin

***Figure S6. Uncropped versions of the western blot used in this manuscript (corresponding to Figure 2 panel B).*** Original gels of Western Blot analysis of Figure 2 panel B: immune-reactive bands corresponding to specific antibodies against Cyclin A, Cyclin D1, p53, p27 and β-actin.

***Supplementary Figure S7***


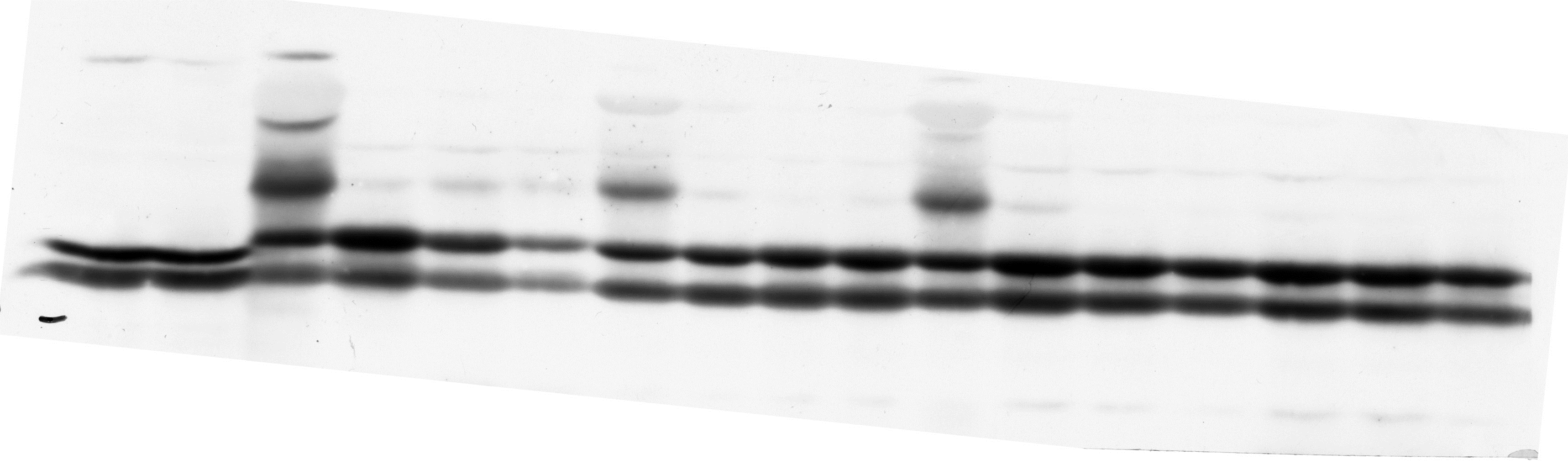
ERK 1/2


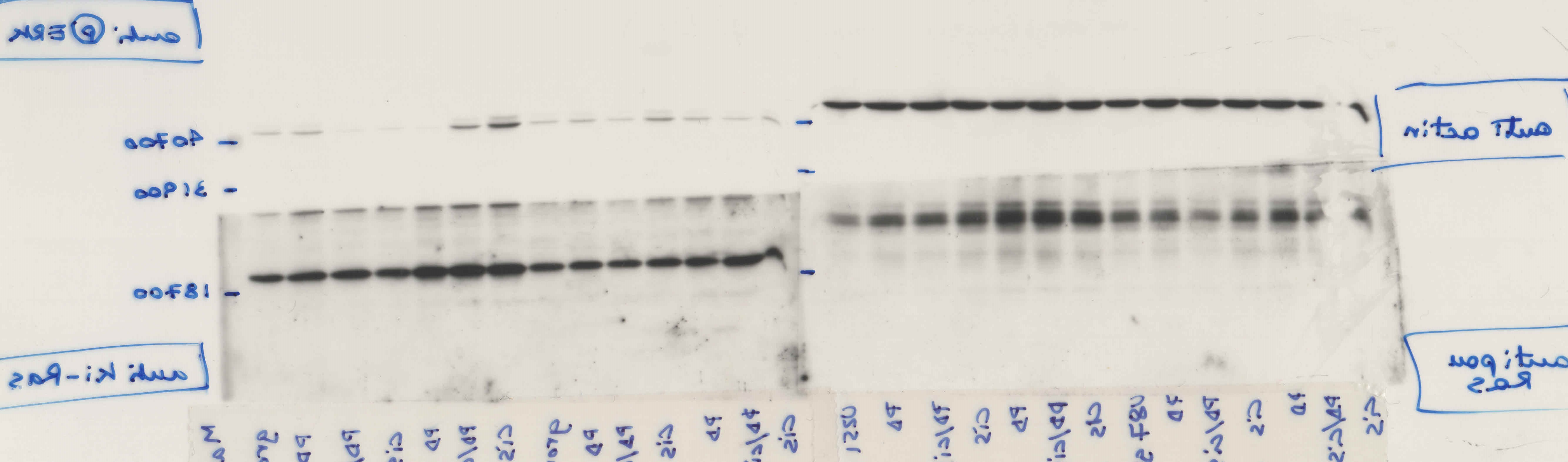


Pan-Ras, K-Ras4B and β-actin


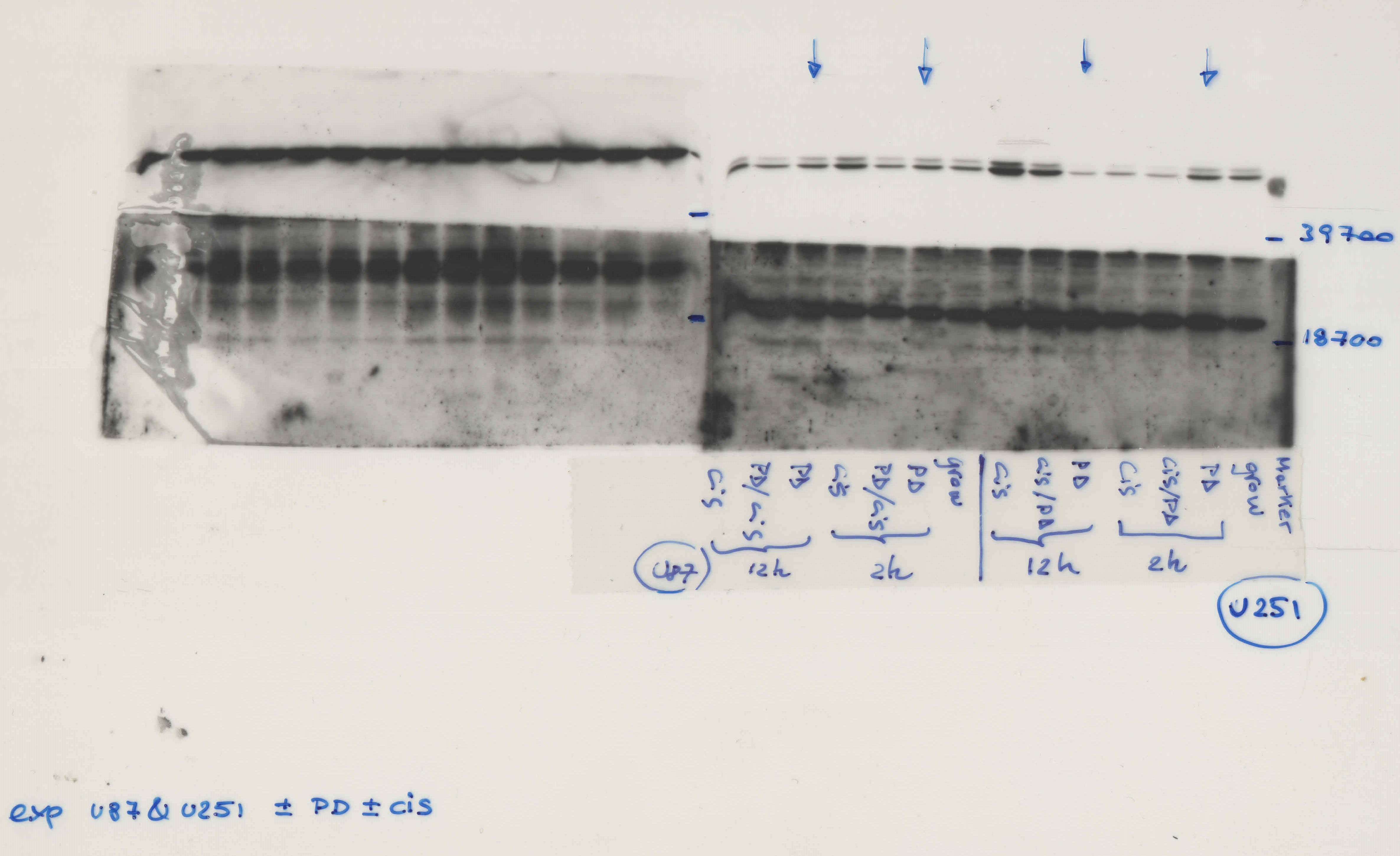


P-ERK 1/2

***Figure S7. Uncropped versions of the western blot used in this manuscript (corresponding to Figure 1bis panel A).*** Original gels of Western Blot analysis of Figure 1bis panel A: immune-reactive bands corresponding to specific antibodies against ERK 1/2, P-ERK 1/2, Pan-Ras, K-Ras4B and β-actin.

***Supplementary Figure S8***


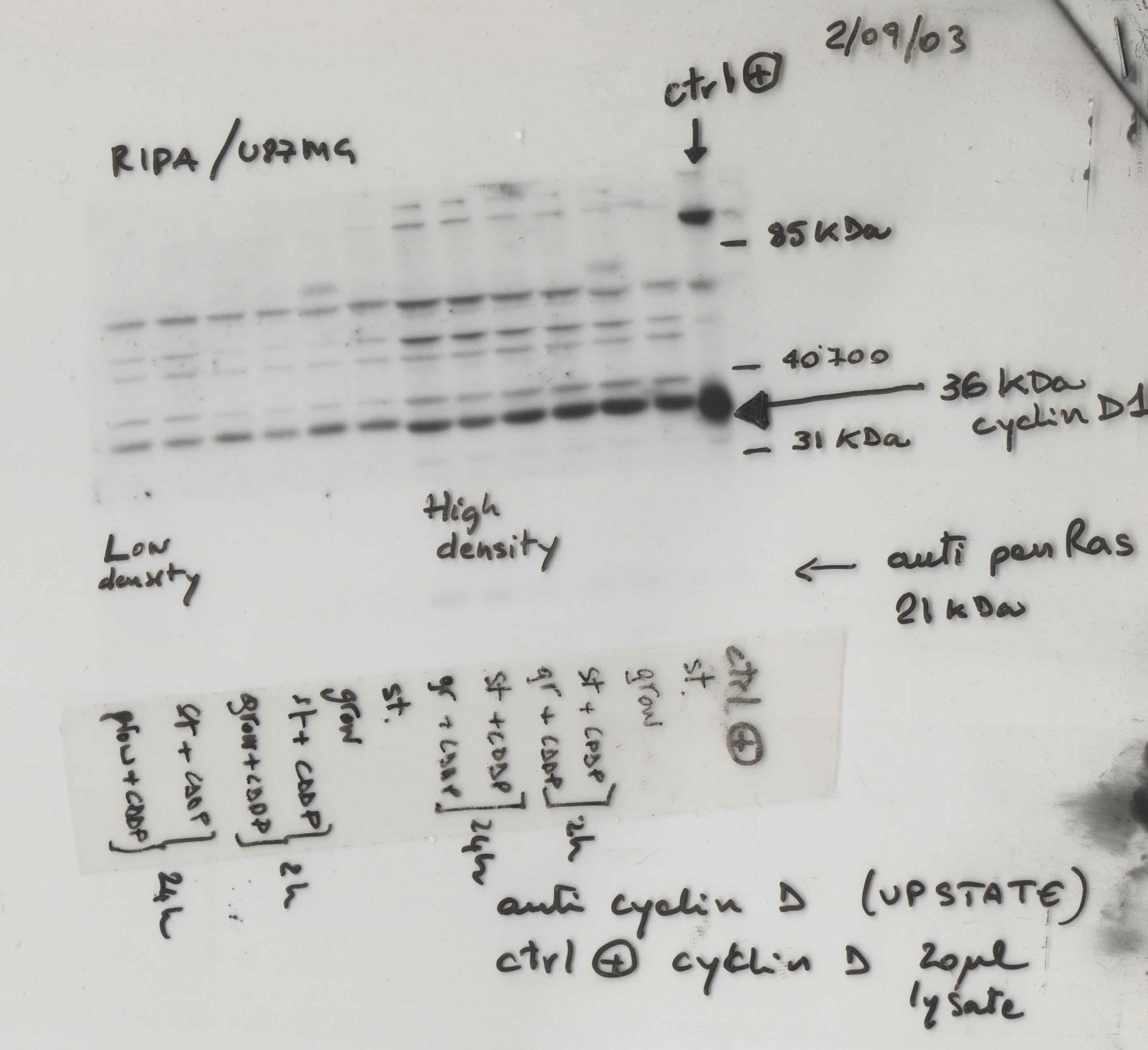
 Cyclin D1

***Figure S8. Uncropped versions of the western blot used in this manuscript (corresponding to Figure S2).*** Original gels of Western Blot analysis of Figure 2S: immune-reactive bands corresponding to specific antibodies against Cyclin D1.

***Supplementary Figure S9***


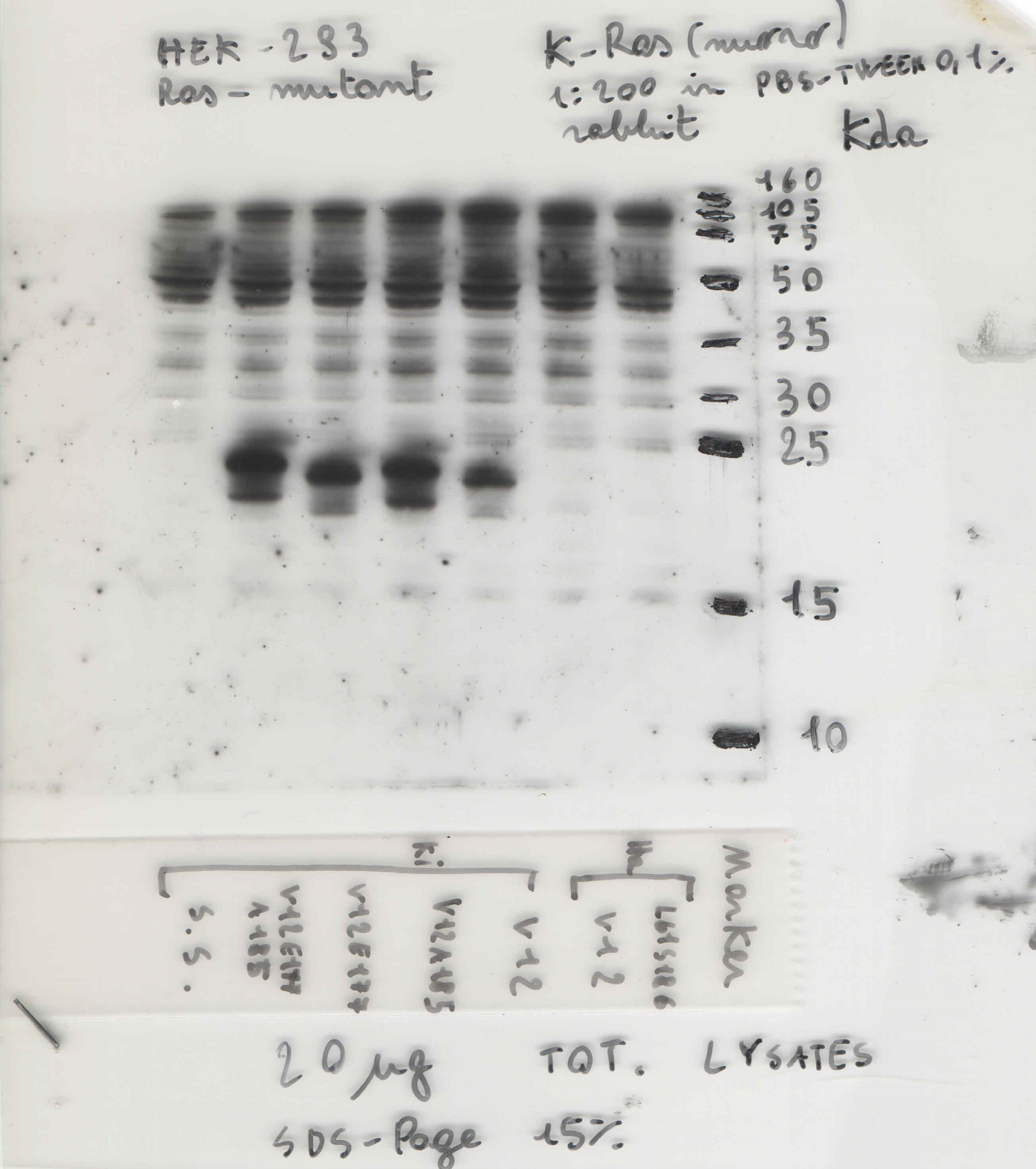
 K-Ras4B

***Figure S9. Uncropped versions of the western blot used in this manuscript (corresponding to Figure S3).*** Original gels of Western Blot analysis of Figure 3S: immune-reactive bands corresponding to specific antibodies against K-Ras4B.
